# Supplementary material for: Did a digital quality of life (QOL) assessment and practice support system in home health care improve the QOL of older adults living with life-limiting conditions and of their family caregivers? A mixed-methods pragmatic randomized controlled trial
Source: PLoS One. 2025 May 6;20(5):e0320306. doi: 10.1371/journal.pone.0320306 (PMC12054893; doi:10.1371/journal.pone.0320306)
Supplement: S2 Table — (DOCX) [file pone.0320306.s002.docx]

S2 Table: Patient sample description

| Characteristics | Total  N = 331 (100%) | Intervention  N = 166 (49.7%) | Control  N = 165  (50.3%) | *p*-value |
| --- | --- | --- | --- | --- |
| Age (n = 331), mean (SD) | 79.1 (9.9) | 79.2 (10.2) | 79.0 (9.7) | 0.80^a^ |
| Gender (n = 331) |  |  |  |  |
| Female | 206 (62.3) | 107 (64.5) | 99 (60.0) | 0.47^b^ |
| Male | 125 (37.8) | 59 (35.5) | 66 (40.0) |  |
| Marital Status (n = 329) |  |  |  | 0.87^c^ |
| Married/Living as married | 105 (31.9) | 52 (31.5) | 53 (32.3) |  |
| Divorced//Separated | 62 (18.8) | 30 (18.2) | 32 (19.5) |  |
| Never married | 33 (10.0) | 15 (9.1) | 18 (11.0) |  |
| Widowed | 129 (39.2) | 68 (41.2) | 61 (37.2) |  |
| Highest level of education (n = 326) |  |  |  | 0.50^c^ |
| Elementary school/Incomplete high school | 64 (19.6) | 31 (19.0) | 33 (20.2) |  |
| High school graduate | 102 (31.3) | 53 (32.5) | 49 (30.1) |  |
| College, trade school, or CEGEP | 92 (28.2) | 50 (30.7) | 42 (25.8) |  |
| University/Post-graduate degree | 68 (20.9) | 29 (17.8) | 39 (23.9) |  |
| Family income per year (n = 280) |  |  |  | 0.28^d^ |
| < $30,000 | 136 (48.1) | 60 (43.5) | 76 (52.4) |  |
| $30,000-$49,999 | 81 (28.6) | 44 (31.9) | 37 (25.5) |  |
| $50,000-$69,999 | 33 (11.7) | 14 (10.1) | 19 (13.1) |  |
| $70,000-$89,999 | 13 (4.6) | 9 (6.5) | 4 (2.8) |  |
| ≥$90,000 | 20 (7.1) | 11 (8.0) | 9 (6.2) |  |
| Ethnic background (n = 316) |  |  |  |  |
| Aboriginal / Indigenous | 8 (2.5) | 3 (1.9) | 5 (3.2) | 0.50^d^ |
| Other North American | 47 (14.9) | 25 (15.7) | 22 (14) | 0.79^b^ |
| British | 126 (39.9) | 63 (39.6) | 63 (40.1) | 1.00^b^ |
| French | 11 (3.5) | 4 (2.5) | 7 (4.5) | 0.38^d^ |
| Western European | 28 (8.9) | 15 (9.4) | 13 (8.3) | 0.87^b^ |
| Southern European | 5 (1.6) | 4 (2.5) | 1 (0.6) | 0.38^b^ |
| Eastern European | 31 (9.8) | 15 (9.4) | 16 (10.2) | 0.97^b^ |
| Northern European | 25 (7.9) | 9 (5.7) | 16 (10.2) | 0.20^b^ |
| European - Other | 5 (1.6) | 4 (2.5) | 1 (0.6) | 0.37^d^ |
| Caribbean | 2 (0.6) | 1 (0.6) | 1 (0.6) | 0.99^b^ |
| Latin, Central, South American | 0 (0) | 0 (0) | 0 (0) | - |
| African | 3 (0.9) | 2 (1.3) | 1 (0.6) | 1.00^d^ |
| East Asian | 8 (2.5) | 5 (3.1) | 3 (1.9) | 0.72^d^ |
| Southeast Asian | 4 (1.3) | 1 (0.6) | 3 (1.9) | 0.39^d^ |
| South Asian | 9 (2.8) | 5 (3.1) | 4 (2.5) | 1.00^d^ |
| West, Central Asian, & Middle Eastern | 2 (0.6) | 2 (1.3) | 0 (0) | - |
| Other Asian | 0 (0) | 0 (0) | 0 (0) | - |
| Oceanian | 2 (0.6) | 1 (0.6) | 1 (0.6) | 1.00^d^ |
| Born in Canada (n = 330) |  |  |  | 1.00^b^ |
| Yes | 242 (73.3) | 122 (73.5) | 120 (73.2) |  |
| No | 88 (26.7) | 44 (26.5) | 44 (26.8) |  |
| Diagnosis for receiving home healthcare (ICD-10 classifications) (n = 291) |  |  |  |  |
| Infectious or parasitic | 5 (1.7) | 2 (1.3) | 3 (2.1) | 0.78^b^ |
| Neoplasms | 25 (8.6) | 14 (9.4) | 11 (7.7) | 0.79^b^ |
| Blood or blood-forming organs | 2 (0.7) | 1 (0.7) | 1 (0.7) | 1.00^b^ |
| Immune system | 5 (1.7) | 3 (2.0) | 2 (1.4) | 1.00^b^ |
| Endocrine, nutrition, or metabolic | 39 (13.4) | 20 (13.4) | 19 (13.4) | 1.00^d^ |
| Mental, behavioural, or neurodevelopmental | 32 (11.0) | 18 (12.1) | 14 (9.9) | 0.68^d^ |
| Nervous system | 78 (26.8) | 39 (26.2) | 39 (27.5) | 0.91^b^ |
| Visual system | 15 (5.2) | 9 (6.0) | 6 (4.2) | 0.66^b^ |
| Ear or mastoid process | 1 (0.3) | 1 (0.7) | 0 (0.0) | 1.00^d^ |
| Circulatory system | 63 (21.6) | 36 (24.2) | 27 (19.0) | 0.36^b^ |
| Respiratory system | 39 (13.4) | 19 (12.8) | 20 (14.1) | 0.87^b^ |
| Digestive system | 20 (6.9) | 11 (7.4) | 9 (6.3) | 0.90^b^ |
| Skin | 2 (0.7) | 0 (0) | 2 (1.4) | 0.24^d^ |
| Musculoskeletal system | 63 (21.6) | 30 (20.1) | 33 (23.2) | 0.62^b^ |
| Genitourinary system | 15 (5.2) | 4 (2.7) | 11 (7.7) | 0.06^d^ |
| Developmental anomalies | 3 (1.0) | 1 (0.7) | 2 (1.4) | 0.62^d^ |
| Symptoms, signs, or clinical findings not otherwise specified | 45 (15.5) | 24 (16.1) | 21 (14.8) | 0.88^b^ |
| Injury, poisoning or other consequences of external cause | 27 (9.3) | 19 (12.8) | 8 (5.6) | 0.06^b^ |
| External causes of morbidity or mortality | 11 (3.8) | 7 (4.7) | 4 (2.8) | 0.52^d^ |

Note. ^a^T-test. ^b^Pearson chi-square with continuity correction. ^c^Pearson chi-square. ^d^Pearson chi-square based on Fisher’s exact test.
