# Supplementary material for: Did a digital quality of life (QOL) assessment and practice support system in home health care improve the QOL of older adults living with life-limiting conditions and of their family caregivers? A mixed-methods pragmatic randomized controlled trial
Source: PLoS One. 2025 May 6;20(5):e0320306. doi: 10.1371/journal.pone.0320306 (PMC12054893; doi:10.1371/journal.pone.0320306)
Supplement: S3 Table — (DOCX) [file pone.0320306.s003.docx]

S3 Table: Family caregiver sample description

| Characteristics | Total  N = 113  (100%) | Intervention  N = 62 (54.9%) | Control  N = 51 (45.1%) | *p*-value |
| --- | --- | --- | --- | --- |
| Age (n = 94), mean (SD) | 66.5 (13.5) | 68.5 (13.6) | 64.0 (13.2) | 0.11^a^ |
| Gender (n = 111) |  |  |  |  |
| Female | 75 (67.6) | 36 (59.0) | 39 (78.0) | 0.06^b^ |
| Male | 36 (32.4) | 25 (41.0) | 11 (22.0) |  |
| Marital Status (n = 110) |  |  |  | 1.00^d^ |
| Married/Living as married | 92 (83.6) | 50 (83.3) | 42 (84.0) |  |
| Divorced//Separated | 10 (9.1) | 5 (8.3) | 5 (10.0) |  |
| Never married | 7 (6.4) | 4 (6.7) | 3 (6.0) |  |
| Widowed | 1 (0.9) | 1 (1.7) | 0 (0) |  |
| Highest level of education (n = 108) |  |  |  | 0.20^d^ |
| Elementary school/Incomplete high school | 5 (4.6) | 5 (8.3) | 0 (0) |  |
| High school graduate | 27 (25.0) | 15 (25.0) | 12 (25.0) |  |
| College, trade school, or CEGEP | 37 (34.3) | 18 (30.0) | 19 (39.6) |  |
| University/Post-graduate degree | 39 (36.1) | 22 (36.7) | 17 (35.4) |  |
| Family income per year (n = 101) |  |  |  | 0.35^d^ |
| < $31,000 | 16 (15.8) | 9 (16.4) | 7 (15.2) |  |
| $31,000 to < $50,000 | 28 (27.7) | 17 (30.9) | 11 (23.9) |  |
| $50,000 to < $70,000 | 22 (21.8) | 8 (14.5) | 14 (30.4) |  |
| $70,000 to < $90,000 | 13 (12.9) | 9 (16.4) | 4 (8.7) |  |
| ≥ $90,000 | 22 (21.8) | 12 (21.8) | 10 (21.7) |  |
| Ethnic background (n = 109) |  |  |  |  |
| Aboriginal / Indigenous North American | 5 (4.6) | 3 (5.0) | 2 (4.1) | 1.00^b^ |
| Other North American | 17 (15.6) | 8 (13.3) | 9 (18.4) | 0.60^d^ |
| British | 35 (32.1) | 18 (30.0) | 17 (34.7) | 0.75^b^ |
| French | 3 (2.8) | 1 (1.7) | 2 (4.1) | 0.59^d^ |
| Western European | 7 (6.4) | 5 (8.3) | 2 (4.1) | 0.46^d^ |
| Southern European | 5 (4.6) | 4 (6.7) | 1 (2.0) | 0.38^d^ |
| Eastern European | 8 (7.3) | 3 (5.0) | 5 (10.2) | 0.46^d^ |
| Northern European | 10 (9.2) | 7 (11.7) | 3 (6.1) | 0.51^d^ |
| European - Other | 5 (4.6) | 2 (3.3) | 3 (6.1) | 0.66^d^ |
| Caribbean | 0 (0) | 0 (0) | 0 (0) | - |
| Latin, Central, and South American | 0 (0) | 0 (0) | 0 (0) | - |
| African | 1 (0.9) | 0 (0) | 1 (2.0) | 0.45^d^ |
| East Asian | 2 (1.8) | 1 (1.7) | 1 (2.0) | 1.00^d^ |
| Southeast Asian | 2 (1.8) | 1 (1.7) | 1 (2.0) | 1.00^d^ |
| South Asian | 2 (1.8) | 1 (1.7) | 1 (2.0) | 1.00^d^ |
| West, Central Asian, & Middle Eastern | 0 (0) | 0 (0) | 0 (0) | 1.00^d^ |
| Other Asian | 1 (0.9) | 1 (1.7) | 0 (0) | 1.00^d^ |
| Oceanian | 1 (0.9) | 1 (1.7) | 0 (0) | 1.00^d^ |
| Born in Canada (n = 111) |  |  |  | 0.73^b^ |
| Yes | 86 (77.5) | 46 (75.4) | 40 (80.0) |  |
| No | 25 (22.5) | 15 (24.6) | 10 (20.0) |  |
| Lives with the patient (n = 111) |  |  |  | 0.67^b^ |
| Yes | 81 (73.0) | 46 (75.4) | 35 (70.0) |  |
| No | 30 (27.0) | 15 (24.6) | 15 (30.0) |  |
| Relationship with patient (n = 111). |  |  |  | 1.00^d^ |
| Patient’s spouse or partner | 60 (54.1) | 33 (54.1) | 27 (54.0) |  |
| Patient’s daughter or son | 40 (36.0) | 22 (36.1) | 18 (36.0) |  |
| Patient’s mother or father | 2 (1.8) | 1 (1.6) | 1 (2.0) |  |
| Patient’s other relative (e.g., daughter-in-law) | 4 (3.6) | 2 (3.3) | 2 (4.0) |  |
| Other (e.g., employee, companion) | 5 (4.5) | 3 (4.9) | 2 (4.0) |  |

Note. ^a^T-test. ^b^Pearson chi-square with continuity correction. ^c^Pearson chi-square. ^d^Pearson chi-square based on Fisher’s exact test.
