# Supplementary material for: Did a digital quality of life (QOL) assessment and practice support system in home health care improve the QOL of older adults living with life-limiting conditions and of their family caregivers? A mixed-methods pragmatic randomized controlled trial
Source: PLoS One. 2025 May 6;20(5):e0320306. doi: 10.1371/journal.pone.0320306 (PMC12054893; doi:10.1371/journal.pone.0320306)
Supplement: S4 Table — (DOCX) [file pone.0320306.s004.docx]

S4 Table: Sample description of qualitative clinician participants

| **Characteristics** | **Clinicians** |
| --- | --- |
| Sample size | 118 |
| Age |  |
| Median | 46.5 |
| Range (min-max) | 25-63 |
| Missing (%) | 19 (16%) |
| Gender |  |
| Female | 111 |
| Male | 4 |
| Other | 0 |
| Missing (%) | 3 (2.5%) |
| Highest Education Level |  |
| College Diploma / Certificate | 32 |
| University (Bachelor) | 61 |
| Graduate School | 22 |
| Missing (%) | 3 (2.5%) |
| Profession |  |
| Registered Nurse | 83 |
| Occupational Therapist | 8 |
| Physiotherapist | 5 |
| Manager | 4 |
| LPN | 7 |
| Social Worker | 7 |
| Missing (%) | 4 (3.4%) |
| Employment Status |  |
| Permanent Full Time | 82 |
| Permanent Part Time | 14 |
| Temporary Full Time | 6 |
| Temporary Part Time | 0 |
| Casual | 4 |
| Years in profession |  |
| Median | 21 |
| Range (min-max) | 1-40 |
| Missing (%) | 4 (3.4%) |
| Years in position |  |
| Median | 2.5 |
| Range (min-max) | 1-33 |
| Missing (%) | 8 (6.8%) |
| Country of birth |  |
| Canada | 73 |
| Asia | 17 |
| Europe | 10 |
| Other | 6 |
| Missing (%) | 12 (10.2%) |
