# Supplementary material for: Did a digital quality of life (QOL) assessment and practice support system in home health care improve the QOL of older adults living with life-limiting conditions and of their family caregivers? A mixed-methods pragmatic randomized controlled trial
Source: PLoS One. 2025 May 6;20(5):e0320306. doi: 10.1371/journal.pone.0320306 (PMC12054893; doi:10.1371/journal.pone.0320306)
Supplement: S5 Table — (DOCX) [file pone.0320306.s005.docx]

S5 Table: Themes and illustrative quotes

| **Action phase** | **Theme** | **Illustrative quote** |
| --- | --- | --- |
| B: ‘adapt knowledge to local context’ | Administrative buy-in of QPSS | “The casual Community Care Professions promptly raised the concern of having the ‘time’ and resources to respond to the gaps, which was cited to be a source of anxiety for the clinicians. She added that clinicians ‘are not (cannot) actively managing the intervention’, citing it as a ‘system issue.’ The manager was prompt to respond to the clinician with ‘we will make time’ for the clinicians, using the QPSS to respond to clients’ concerns.”  [**q1,** Pre-intervention period, focus group field note, Sites 1 & 2] |
|  |  | “The team has not stopped meeting bi-monthly since the [pre-intervention period began 1 year ago.] Responding to my observation, the clinician said that the clinicians knew ‘it’s QPSS time’ when the team leader approached them in person before the meetings.”  [**q2,** Pre-intervention period, focus group field note, Site 5] |
|  | Complementarity of workflow & other data | “So, when you look at RAI [Resident Assessment Instrument], sometimes it doesn’t really give you - the RAI is more numeric to some degree. But this one is more - there’s quantitative data, but still the questions are more about qualitative data, but this put in numbers. And then when you do the interview you can ask them. ‘So, your answer in this one and this is what - so what do you mean by this?’”  [**q3,** Pre-intervention period, focus group, Site 3] |
|  |  | “We have to live within our home support guidelines. And, so my concern is when we’re talking about a client or a caregiver who’s having a lot of extra stress, et cetera… I mean, this information is great, but there’s only so much that we’d be able to do with it. As long as clients don’t expect us to fix it, right. So, it allows us to reflect on their responses as well, right and, like, our practice and the services that we’ve put in.”  [**q4,** Pre-intervention period, focus group, Site 4] |
|  |  | “The team leader commented that the data collected via the QPSS will be a ‘gold mine’ for interdisciplinary team consultation meetings, bringing awareness to clinicians the things that matter to the clients.”  [**q5,** Pre-intervention period, focus group field note, Site 5] |
| C: ‘assess barriers to knowledge use’ | Strategic data display | “When you search for their actual questionnaires, you can’t search by the client’s name. You have to go into ‘search’ and then type in a date range and then pull up all the assessments that are done for your whole group… There might be hundreds of questionnaires that have been done. You have to pick through and find your client.”  [**q6,** Pre-intervention period, interview, Site 4.]   - Note: in response, a search function was built into the QPSS |
|  |  | “[A staff member] was at the meeting to present the QOL results reporting feature and to seek the clinicians’ feedback.”  [**q7,** Pre-intervention period, interview, Site 2] |
|  |  | “A clinician expressed the value of printing off the profile graphs and taking them with her to home visit. The documents serve as a talking point during interaction with clients.”  [**q8,** Pre-intervention period, interview, Site 5] |
|  | Sufficient time, resources, and capacity | “The social worker expressed concern that they may not be able to meet the needs identified through the questionnaires due to the limited resources in the system.” [2 months later in a subsequent focus group] “The project manager/team leader reminded that the QPSS would be a tool for clinicians to use, taking the burden off the clinicians when they are informed to make referral of services to the clients.”  [**q9,** Pre-intervention period, focus group field note, Site 2] |
|  |  | “Clinical practice lead raised the issue of the obligation for prompt response to a need flagged in the assessment, and that they would have to ‘drop everything’ to respond to a highly distressed situation. Team leader responded [that] distressed clinicians could go to her.”  [**q10,** Pre-intervention period, focus group field note, Site 7] |
| D: ‘tailor and implement intervention’ | Appropriateness of QOL assessment tools | “Have had 4 focus groups to date, the most recent to determine choice of tools to be made accessible to clinicians in the QPSS and workflow integration. On tool selection:   - Identified subjective questions on broader range of QOL domains, like MQOL and QOLLTI, are more helpful than the symptoms specific questions, as in ESAS - Some parts of CANHELP questions on satisfaction with care are helpful, but not those related to physicians’ care.”   [**q11,** Pre-intervention period, focus group field note, Site 1] |
|  | Importance of clinical judgement for use of QOL assessment data to inform practice | “The [nurse] would be able to review the questionnaires and use their own clinical judgement to review the assessment with the client or caregiver and help guide some of their questions. For clients, for example, who [need] a home visit because there's a change in their condition now, I'm not sure how that would work because then we would go out and do the RAI assessment, and then would they do this assessment as well? I'm not sure.”  [**q12,** Pre-intervention period, interview, Site 4] |
|  |  | “I think the cueing is probably going to be big – for [the patient or family caregiver] to say, ‘Well, maybe I can talk to them [clinician] about this.’”  [**q13, ­**Pre-intervention period, interview, Site 5] |
| E: ‘monitor knowledge use’ | Routine use of the QPSS by home in practice | “I didn’t realize you could print [the QOL assessment results]. I never really looked, to even think about doing that.”  [**q14,** Intervention period, focus group, Site 5] |
|  |  | “One of the nurses wanted to look at the trends and a different nurse said she had never used it before. But they all agreed it was very helpful and probably more helpful that what they had been doing before.”  [**q15,** Intervention period, focus group field notes, Sites 8 & 9] |
|  |  | **Negative case analysis** example of a clinician using the QOL information after viewing it on QPSS:  Clinician: “Have someone interpret [the questionnaire] … the nurse will follow up and call the patient and the family to get more info, more history. So that when they talk to the doctor, it’s not a back and forth….It just speeds it up.  Interviewer: “Would there be any way you would know if an issue that you brought up based on these assessments got addressed during the clinic visit?”  Clinician: “Yeah. Everyone will chart after they’ve spoken, or anything you do with a patient - we’ll chart it in a visit note. So yeah, you could check, make sure they talked to them about it, what they said and then if there was something you wanted to follow up on, you could do that.”  [**q16,** Intervention period, interview, Site 9] |
|  | Competing priorities | Clinician: “We don’t have administrative support. So, the amount that we have to do, that we would never have to do in a doctor’s office, and have things flagged for us and follow up - that piece is so cumbersome. The [quality of life assessments] in Cambian are low on my priority list to access at the end of the day because - we send our own referrals. When an [electronic medical record] system is, like, ‘okay, send that there,’ like somebody else does that grunt work of it. Whereas, I find that our [electronic medical record] system is so cumbersome and time consuming for myself in a day. And that then –  Clinician: You forget about it.  Clinician: Yeah. I think that’s right. When you don’t see it regularly.  Clinician: Yeah, that’s true.  [**q17,** Intervention period, focus group, Sites 8 & 9] |
|  |  | **Negative case analysis** example of a clinical lead seeing use of QOL information as integral, in other words, not a competing priority: “… a clinician asked whether their participation was voluntary. The clinical lead explained that their participation in this aspect of the study i.e., reviewing client and family caregiver quality of life assessments, was not voluntary. Their participation in future focus group sessions and individual interviews, is voluntary.”  [**q18,** Intervention period, focus group field note, Site 4] |
|  | System integration | “If it was attached to one of our platforms, I don’t know, there might be a chance that we would just kind of go in there and click, because it’s right there…Maybe you would see it visually and might access it. The likelihood of accessing it may increase.”  [**q19,** Intervention period, focus group, Site 2] |
|  |  | **Negative case analysis** example of not perceiving the QPSS as a barrier because it was on a separate website:  Clinician 1: “It’s pretty straightforward to access.”  Clinician 2: “It was very easy.”  Clinician 1: “I mean, it’s just like logging on in the morning. So you have to log on to [electronic medical record #1] and – “  Clinician 2: “[electronic medical record #2].”  Clinician 1: “[electronic medical record #2], so we would just log on to [the QPSS] and just… as part of the routine and print out if we needed it, to bring to the client’s home and that sort of thing.”  [**q20,** Intervention period, focus group, Site 5] |
|  | Technological challenges | “Our visits are not on-site. They’re at the patients’ homes…a lot of our computers are aged and they’ve been recycled, and so we have to use our hotspot on our phones, but by the time you try to log…you can’t even get on. Like it takes 20 minutes and you’re closed out.”  [**q21,** Intervention period, focus group, Sites 8 & 9] |
|  |  | **Negative case analysis** example of workarounds by printing QOL information or viewing this information on a laptop: “Sometimes, I have to print the paper off. I find it hard to read on the computer. And then to show the patient, they want to see the copy and they want to look at the results. So, it would be probably handier to have a laptop to show them visually. But then it’s nice to have a take home piece too.”  [**q 22,** Intervention period, focus group, Sites 8 & 9] |
|  | Time constraints | “To be honest, I’ve had a little bit of a hard time using [the QPSS] just because my caseload has been so large that incorporating that into my practice has taken a little bit of a toll, just because there’s so much going on. And then also incorporating the home care nursing aspect that they’ve now enrolled with us. So, it’s nothing to do with the system itself. It’s just incorporating it into it has just been a little bit difficult, yeah. Just a lot on my plate.”  [**q23,** Intervention period, joint focus group with Sites 2, 3, 4, 5 & 6) |
|  |  | **Negative case analysis** example of the importance of integrating QOL information and it not taking more time: “If you can engage or integrate the quality of life part more – you integrate it more in-depth – I think that – There’s always tasks to be done. But I think it, again, makes it a wider breadth of care that you’re able to provide at that point because it’s just not all about tasks. There’s so much more to that psychosocial component.”  [**q24,** Intervention period, interview, Site 9] |
