# Supplementary material for: Did a digital quality of life (QOL) assessment and practice support system in home health care improve the QOL of older adults living with life-limiting conditions and of their family caregivers? A mixed-methods pragmatic randomized controlled trial
Source: PLoS One. 2025 May 6;20(5):e0320306. doi: 10.1371/journal.pone.0320306 (PMC12054893; doi:10.1371/journal.pone.0320306)
Supplement: S1 Protocol — (PDF) [file pone.0320306.s006.pdf]

Integrating a quality of life assessment and practice support system in homecare services for older adults with life-limiting illness and their families

Study Protocol
Version 19
January 12, 2018

Note: This amendment pertains to the randomized control trial (RCT) and process evaluation in Stage 2 of the project (highlighted in yellow font throughout). The amendment was approved prior to commencing data collection for the RCT. The original REB application involved only the focus groups and previous amendments focused only on Stage 1 of the project. The economic evaluation component of Stage 2 and the sustainability study in Stage 3 will be detailed in future amendments and are only briefly described herein so that it is clear how each stage relates to the overall project.

Principal Investigator:
Dr. Richard Sawatzky, Professor, School of Nursing, Trinity Western University

Co-Principal Investigators:
Dr. Robin Cohen, Professor, Departments of Oncology and Medicine, McGill University
Dr. Kara Schick-Makaroff, Assistant Professor, Faculty of Nursing, University of Alberta
Dr. Kelli Stajduhar, Professor, Centre on Aging and School of Nursing, University of Victoria

Principal Knowledge User:
Dr. Neil Hilliard, Program Medical Director, End of Life Care, Fraser Health Authority

Decision maker:
Ms. Lisa Zetes-Zanatta, Executive Director New Westminster and Tri-Cities Communities, Eagle Ridge Hospital, Centralized Home Health Services, Regional Home Health Network, Fraser Health Authority

TABLE OF CONTENTS

1 STUDY TEAM MEMBERS....4
2 STUDY PROTOCOL SUMMARY....6
3 INTRODUCTION AND BACKGROUND....7
3.1 eHealth Innovation Enabled Care Delivery Program....9
3.2 Intellectual property....10
3.3 Quality of Life and Practice Support System (QPSS)....10
4 JUSTIFICATION....11
4.1 Expected Outcomes....11
5 PURPOSE....11
6 METHODS....12
6.1 Overview of Research Design....12
6.1.1 Stages of the research project....13
6.1.2 Experimental design....14
6.2 Methods for Stage 1: Local integration and exploratory use of the QPSS intervention....14
6.2.1 Development of the QPSS implementation protocol....14
6.2.2 Data collection from the pre-intervention comparator group....14
6.2.3 Transition phase to explore use of the QPSS intervention....15
6.3 Methods for Stage 2: Evaluation of outcomes, cost-effectiveness and processes of use....15
6.3.1 The QPSS intervention....15
6.3.2 Intervention group....15
6.3.3 Control group....16
6.3.4 Allocation to intervention and control conditions....16
6.3.5 Concealment of group allocation....16
6.3.6 Contamination....16
6.3.7 Assessing adherence....17
6.3.8 Quantitative data collection (questionnaires)....17
6.3.9 Data collection from health records....17
6.3.10 Qualitative data collection (focus groups and interviews)....17
6.4 Sampling Design and Subject Selection....18
6.4.1 Clinicians....18
6.4.1.1 Inclusion criteria....18
6.4.1.2 Exclusion criteria....18
6.4.1.3 Stage 1....18
6.4.1.4 Transition phase....18
6.4.1.5 Stage 2....18
6.4.2 Patients and family caregivers....18
6.4.2.1 Inclusion criteria....18
6.4.2.2 Exclusion criteria....19
6.4.2.3 Stage 1....19
6.4.2.4 Transition phase....19
6.4.2.5 Stage 2....19
6.5 Recruitment and Informed Consent....19
6.5.1 Clinicians....19
6.5.1.1 Stages 1 and 2....19
6.5.2 Patients and family caregivers....20
6.5.2.1 Stage 1....20
6.5.2.2 Transition phase....21

6.5.2.3 Stage 2 22
6.6 Data Collection 23
6.6.1 Clinician focus groups and interviews 23
6.6.1.1 Stage 1 and transition phase 23
6.6.1.2 Stage 2 23
6.6.2 Patient and family caregiver outcome evaluation data 23
6.6.2.1 Stages 1 and 2 23
6.6.2.2 How patients and family caregivers may choose to use the Navigator system 24
6.6.3 Patient and family caregiver interview data 24
6.6.4 Patient and family caregiver administrative data 25
6.6.5 Data regarding use of the Cambian applications 25
6.7 Use of the QPSS in practice during the transition phase and Stage 2 25
6.8 Outcome Measures 26
6.8.1 Primary outcome measures 26
6.8.2 Secondary outcome measures 26
6.8.3 Other measures 26
6.9 Analytical Plan 26
6.9.1 Analysis of clinician focus groups and interviews and patient/family caregiver interviews 26
6.9.2 Analysis of outcomes data 27
6.9.2.1 Sample size justification 27
6.9.3 Analysis of administrative data in cost-effectiveness evaluation 27
6.10 Potential Benefits 28
6.11 Potential Risks 28
6.11.1 Patients and family caregivers 28
6.11.2 Clinicians 28
6.12 Incidental findings 29
6.13 Subject Safety Provisions 29
6.14 Data Security and Protection of Privacy 29
6.14.1 Focus group (clinician) and interview (clinician, patient, family caregiver) data 29
6.14.2 Patient and family caregiver QPSS data 30
6.14.3 Data Access 30
6.14.4 Storage of paper and audio data 31
6.15 Ethics Approval 31
7 PLANS FOR PUBLICATION AND CONFERENCE PRESENTATIONS 31
8 TIMELINE 31
9 REFERENCES 32

1 STUDY TEAM MEMBERS

Core Leadership Team
Principal Investigator
Dr. Richard Sawatzky, Professor, School of Nursing, Trinity Western University

Co-Principal Investigators
Dr. Robin Cohen, Professor, Departments of Oncology and Medicine, McGill University
Dr. Kara Schick-Makaroff, Assistant Professor, Faculty of Nursing, University of Alberta
Dr. Kelli Stajduhar, Professor, Centre on Aging and School of Nursing, University of Victoria

Principal Knowledge User
Dr. Neil Hilliard, Program Medical Director, End of Life Care, Fraser Health Authority

Decision maker
Ms. Lisa Zetes-Zanatta, Executive Director New Westminster and Tri-Cities Communities, Eagle Ridge Hospital, Centralized Home Health Services, Regional Home Health Network, Fraser Health Authority

Patient and Family Engagement Panel
Ms. Shelly Cory, Executive Director, Virtual Hospice Executive Team
Ms. Barbara McLean, Executive Director, Family caregivers of British Columbia
Mr. Geoff Cowman, Chair, Canadian Association of Retired Persons Richmond/Delta Chapter
Ms. Mary Luk, Family Caregiver
Ms. Alies Maybee, Patient/Family Caregiver
Ms. Jennifer Huang, Patient

Clinician and Healthcare Administration Panel
Clinicians
Ms. Susan Brown, Clinical Nurse Specialist, Fraser Health Authority
Mrs. Barbara McLeod, Clinical Nurse Specialist, Fraser Health Authority
Ms. Della Roberts, Clinical Nurse Specialist, Island Health
Ms. Madeleine Fraser, Home Care Nurse, Alberta Health Services
Ms. Kya Milne, Palliative Nurse, Fraser Health Authority

Healthcare Administration
Ms. Lena Cuthbertson, Provincial Director, British Columbia Ministry of Health
Mrs. Jill Gerke, Regional Manager, Island Health Authority
Ms. Cori Paul, Advanced Practice Manager, Alberta Health Services
Ms. Carolyn Tayler, Investigator, Initiative for a Palliative Approach in Nursing: Evidence and Leadership
Ms. Daphne Williscroft, Interim Director, Fraser Health Authority
Mrs. Sharon Baxter, Executive Director, Canadian Hospice Palliative Care Association

Health Technology Panel
Dr. Bruce Forde, CEO, Cambian Business Services
Dr. Herbert H. Tsang, Professor, Department of Computing Science and Mathematics, Trinity Western University
Mr. James Voth, Founder and Director, Intogrey Research and Development Inc.

Researcher Panel

Canadian researchers:

Dr. Joan Bottorff, Professor, School of Nursing, University of British Columbia
Dr. Stirling Bryan, Professor, School of Population and Public Health, University of British Columbia
Dr. Peter Dodek, Professor, Critical Care Medicine, University of British Columbia
Dr. Kimberly Fraser, Associate Professor, Faculty of Nursing, University of Alberta
Dr. Anne Gadermann, Post-doctoral fellow, Centre for Health Evaluation and Outcomes Sciences, St. Paul's Hospital, University of British Columbia
Ms. Esther Laforest, Doctoral nursing student, Ingram School of Nursing, McGill University
Dr. Sheryl Reimer-Kirkham, Professor, School of Nursing, Trinity Western University
Dr. Pamela Ratner, Professor, School of Nursing, University of British Columbia
Dr. Lara Russell, Post-doctoral fellow, School of Nursing, University of Victoria
Dr. Maria Santana, Assistant Professor, University of Calgary, Associate Director Strategy for Patient-oriented Research (SPOR) Methods Platform, Alberta CIHR Strategy for Patient-oriented Research
Dr. David Whitehurst, Assistant Professor, Faculty of Health Sciences, Simon Fraser University

International researchers:

Dr. Todd Edwards, Research Assistant Professor, Department of Health Services, University of Washington
Dr. Gail Ewing, Senior Research Associate, Cambridge University
Professor Gunn Grande, University of Manchester
Professor Joakim Öhlén, Institute of Health and Care Sciences, University of Gothenburg

2 STUDY PROTOCOL SUMMARY

This project brings together researchers, two industry partners, multiple clinical partners, health care administrators, and patients and family caregivers who will conduct collaborative research to enhance patient and family caregiver quality of life (QOL) while improving the efficacy of the health care system by studying the implementation and integration of an innovative health care information system, the Quality of life Assessment and Practice Support System (QPSS), into routine care provided by home care services for older adults who have life-limiting illnesses and their family caregivers. The QPSS includes carefully selected and validated questionnaires that clinicians can use to obtain patients' and family caregivers' perspectives about their QOL and health care needs and intervene appropriately. Patients, family caregivers, and clinicians will all use the QPSS during home visits as a routine part of the care provided. Administrators and other decision-makers will use the entire information system to improve care at all levels of the health care delivery system, ensuring that resources are used effectively.

Research aims for the overall project

AIM 1: To examine the efficacy of the QPSS in home care for older adults who have advancing life-limiting illnesses.

AIM 2: To evaluate the cost consequences of implementing the QPSS in home care.

AIM 3: To understand the process of implementing the QPSS in home care.

AIM 4: To examine how integration of the QPSS with health information systems will meet the needs of administrators for quality improvement and accreditation.

Design of the overall project

This project will be conducted in 3 stages at 7 home care service sites.

STAGE 1: The **local adaptation stage** will use qualitative methods (user-centered design focus groups and interviews with clinicians, managers, patients, and family caregivers) to adapt the QPSS to the unique context of each site and develop protocols for its routine integration in daily practice. To test the recruitment and data collection procedures and explore the possibility of contamination and the potential impact of changes in the health care system between Stages 1 and 2, this will also include (a) measurement of patient and family caregiver QOL and health care experiences prior to routine use of the QPSS in home care to serve as a pre-intervention comparator group (Aim 1) and (b) data collection regarding the cost of local adaptation and training (Aim 2).

TRANSITION PHASE: In the last 6 months of Stage 1, a few clinicians from each site will explore the use of the QPSS with 1 to 2 of the clients in their respective caseload. The clinicians trying out the QPSS will provide feedback on their experience using the system via focus group (in Stage 1) and through one-time individual interview. Clients and family caregivers who tried out the QPSS will also be interviewed. The feedback from clinicians, clients and family caregivers will inform the development of the site protocol, as well as the preparation for QPSS implementation in Stage 2.

STAGE 2: During the **evaluation stage**, we will (a) conduct a randomized controlled trial (RCT) to evaluate the impact of using the QPSS on the QOL and care experiences of patients and their family caregivers (Aim 1); (b) conduct an economic evaluation to estimate the cost consequences of the QPSS in home care (Aim 2) in the last six months of Stage 2; and (c) apply qualitative and quantitative methods to examine the use of the QPSS in home health care (Aim 3).

STAGE 3: The **sustainability stage** will focus on (a) whether the results warrant sustained and widespread integration of the QPSS into practice, and if so, (b) how to scale this up, (c) how to further integrate the QPSS with other health information systems to facilitate use by health care administrators for continuous quality improvement and accreditation (Aim 4) and (d) dissemination of results.

We will use qualitative methods (user-centered design focus groups with clinicians and managers, and interviews with patients and family caregivers) to adapt the QPSS to the unique context of each site and develop protocols for its routine integration in daily practice. For evaluative purposes, we will administer

questionnaires to patients and family caregivers to obtain baseline measurements of their QOL and health care experiences as a pre-intervention comparator group for the outcomes evaluation in stage 2 of the project (Aim 1). In addition, we will collect data about the cost of local adaptation and training (Aim 2).

The current REB amendment pertains to the randomized control trial (RCT) in Stage 2 of the project. The Fraser Health home health offices enrolled included New Westminster, Tri-Cities, White Rock, Gateway, South Delta, and Burnaby (see Appendix C). Privacy impact assessment has been conducted by the Fraser Health Privacy Office. The project has also obtained approval from Alberta Health Services REB to commence in the Edmonton Zone Home Living sites. Future amendments for research ethics and approval will be submitted for the cost-consequences evaluation and the sustainability (Stage 3) aspects of the project. Island Health sites are withdrawn from the project. They will no longer be enrolled in the project, and no further amendments following the approval for focus group (Stage 1) is required. Sections pertaining to Island Health in version 10 (October 12, 2016) of the protocol are removed from the current protocol. The numbering of all currently approved appendices will remain unchanged.

3 INTRODUCTION AND BACKGROUND

The rising number of people facing old age makes it likely that advancing chronic and life-limiting illness will be a dominant challenge for health care delivery in the next half-century. Older adults (for this project, people ≥ 55 years old) who have an incurable and advancing life-limiting illness[1] often choose to be cared for at home in order to live their last years and months as fully and as comfortably as possible. This requires health care that focuses on improving their and their family caregivers' quality of life (QOL) through early identification, assessment, and treatment of their multiple complex symptoms and concerns in a wide range of life domains: physical, psychosocial, and spiritual.[1] Comprehensive, ongoing reporting of QOL and health care concerns by the patients and family caregivers themselves, and action based on these reports by home care clinicians, are required to inform patient- and family-centred approaches to care planning and decision making to improve quality of care and enhance QOL. One of the imperatives for person-centred rather than disease-centred care is taking into account that concerns in one life domain impact the others. For example, bodily pain can be increased by spiritual or psychological distress and *vice versa*, and the most effective pain management involves addressing these sources of distress along with interventions aimed directly at reducing pain.[2] **The proposed study enables best practice for this population with complex needs by studying the implementation and integration of an electronic innovation, the Quality of life Assessment and Practice Support System (QPSS), into routine care provided by home care services for older adults with life-limiting illness and their family caregivers.**

Home care clinicians (nurses, nurse practitioners, social workers, physicians, and other allied health care professionals) play a key role in providing care at home to people who have advancing, chronic, and life-limiting illnesses. In this role, they aim to ensure that the full range of health care needs relevant to the QOL of their patients and that of their family caregivers[2] are recognized and adequately addressed. This includes assessments not only of patients' symptoms, but also of other aspects including their physical,

---

[1] **An advancing life-limiting illness** refers to conditions that are progressing, for which there is no cure and that can be expected to cause death, inclusive of malignant and non-malignant illnesses. Examples include advancing chronic obstructive pulmonary disease, heart disease, kidney disease, diabetes, dementia, and general frailty. We are focusing on people with advancing chronic, life-limiting illnesses who may or may not currently be receiving palliative home care services. However, all require care aimed at improving their QOL by preventing and relieving suffering through early identification, assessment, and treatment of physical, psychosocial, and spiritual concerns.

[2] **Family caregivers** are identified by patients and may include caregivers who are "lay people in a close supportive role who share in the illness experience of the patient and who undertake vital care work and emotion management".[3. National Institute for Clinical Excellence, *Guidance on cancer services: Improving supportive and pallaitive care for adults with cancer: The manual.* 2004, NICE: London.

psychological, social and existential wellbeing that are important to their QOL.[4, 5] In addition, routine assessments of family caregivers' QOL are required to prevent their own health from worsening (many are seniors with their own health issues) and enable them to provide care both at home and for a longer duration. Family caregivers often work closely with the home care team and, together, assume major responsibility for the coordination and delivery of care.[6, 7] Indeed, patients' ability to be cared for at home is heavily dependent on the efforts of family caregivers.[8] Though normally done willingly,[9] the work of family caregivers in caring for someone who has a life-limiting illness at home comes at significant emotional, social, financial and physical cost, and even increased mortality.[10, 11] While there is a clear need to ensure that the QOL concerns and supportive needs of family caregivers are also routinely assessed and addressed by home care clinicians, this aspect of care is often neglected.[12]

Use of standardized self-reported QOL assessment instruments,[3] including *patient-reported outcome measures* (PROMs) and *patient-reported experience measures* (PREMs), **integrated with other clinical practice supports[4]** into routine home care, can enable clinicians to efficiently assess and address the needs and concerns of patients and family caregivers and fluctuations in their QOL including their symptoms, functional status, psychological, social, and existential wellbeing, as well as their experiences with the care provided.[14-17, 19, 24] Primary studies and systematic reviews have shown that providing clinicians with this kind of information can improve clinician-patient communication, raise awareness of problems that would otherwise be unidentified, improve care plans, and improve multidisciplinary collaboration.[16, 20, 25-35] Health care administrators and managers increasingly advocate for the routine use of PROMs and PREMs because of their potential to improve patient care and reduce costs by helping clinicians to work more efficiently, enabling them to more quickly and accurately assess and address patient problems as soon as they arise. In addition, information from PROMs and PREMs is increasingly used for program evaluation and quality improvement from a patient-centered care point of view.[36-38] Although the use of similar information about the QOL and health care experiences of family caregivers has been less studied, it is reasonable to expect that this information would facilitate improvement of caregivers' wellbeing and potentially enhance their ability to continue providing care, especially given that they are typically reluctant to mention their own needs without prompting.[9, 39]

Despite the widely-recognized need to pay attention to patients' and family caregivers' self-reports about their QOL and health care needs and the availability of many QOL assessment instruments, the integration of these assessment instruments into the daily practice of home care providers has been elusive. Many QOL assessment instruments are paper-based and not easily integrated into comprehensive information systems. Shortcomings of previous studies in integrating PROMs into practice include: 1) a lack of consultation with clinicians about the design of the tool, 2) paucity of information about how to integrate them into practice, as well as 3) clinicians not wanting to measure outcomes they feel ill-prepared to address.[14, 25, 29, 30, 33]

---

[3] We use the term "**QOL assessment instruments**" to be inclusive of **patient-reported outcomes measures** (PROMs) and **patient-reported experience measures** (PREMs) that are used to assess the QOL of patients and family caregivers. Patient-reported outcomes measures (PROMs) refer to self-report instruments used to obtain appraisals from health care recipients about outcomes relevant to their health and QOL. Many PROMs are multidimensional in nature in that they measure experiences, needs, and satisfaction in various life domains, including those related to symptoms, functional status, health status, and psychological, social, and spiritual wellbeing. (13-20) Patient-reported experience measures (PREMs) include measures of satisfaction and experiences with the care provided. (21-23) Although the term "patient" is conventionally used to refer to these measures, we mean it to refer to all recipients of health care, including family caregivers. (24)

[4] Other clinical practice supports include simultaneous tracking of QOL concerns, interventions used to address concerns over time, and local practice guidelines and procedures.

User-centered electronic information systems, made accessible at the point of care, are recommended to facilitate the integration of self-reported QOL assessment instruments in practice.[24, 34, 40-47] For the integration of such systems to be successful, it is imperative that the end users (clinicians, patients, family caregivers) be closely involved in their design, development, implementation, evaluation, and modification.[14, 48-50] The optimal system must be user-friendly, deliver relevant and timely access to information at the point of care, be well-integrated into established workflows, include mechanisms that facilitate care planning and decision-making processes, and be adaptable to the needs of all users. At the same time, the system must be integrated with other health information systems so that patient and family caregiver information can be used for program evaluation and health system performance monitoring. This is a complex intervention.[51]

Gaps. Various electronic assessment tools have been studied with a focus on particular aspects of care,[41-43, 52-55] such as symptom management (e.g., *Edmonton Symptom Assessment System*). However, the studies of people with life-limiting illnesses have predominantly focused on oncology patients, and there has been little emphasis on interdisciplinary teams, family caregivers' QOL, and patient- and family-reported experiences with the care provided (PREMs).[56, 57] There is also a lack of knowledge about how to best integrate such systems into the routine practice of interdisciplinary health care teams, particularly in home care settings, and whether such routine assessment will make a difference for these home care patients and their family caregivers in terms of their QOL and quality of care. In addition, health care administrators require information about the cost-effectiveness of such systems and their integration with existing information technologies for quality improvement and health services evaluation.

3.1 eHealth Innovation Enabled Care Delivery Program

Over the past five years, our team of researchers, clinicians, and health care administrators has worked closely with a software developer to design a secure, integrated health care information system that facilitates point-of-care data collection via computer tablets (using a web-based application) and the use of information from patients and their family caregivers regarding their perceived health outcomes and health care experiences. The QPSS was developed in the context of two catalyst projects, funded by the Canadian Frailty Network (formerly Technology Evaluation in the Elderly Network), an NCE-funded network. For these projects, we conducted user-centered design focus groups and interviews with palliative care clinicians and administrators to inform the QPSS development. We also interviewed patients and family caregivers regarding their experiences with use of the QPSS as part of their care (see Appendix 1). Specifically, health care providers explained the features of the feedback system that were desirable to them. Suggested revisions to the QPSS included how results could be disseminated in real-time at point of care, how results were visually displayed, how to potentially link with clinical practice guidelines, and how PROM and PREM data could be integrated with electronic charting to prevent duplication. Patients' and family caregivers' feedback guided refinement of the directions about how to use the system and offered insights about how they would like their needs and concerns to be addressed by their health care providers.

Partnership. The project is funded by the CIHR electronic Health Information Partnership Program which has the mandate "to facilitate experimental "real-world" large scale implementations, focusing on the integration of existing innovations to create value (effectiveness, efficiency, cost-savings) by developing inter-sectoral collaboration between: health care innovation communities including researchers, clinicians, patients, decision makers, and industry" (http://www.cihr-irsc.gc.ca/e/48614.html). The relationships with partners has been established in accordance with the CIHR criteria.

The research is supported by a standard commercial agreement for the supply of specific software and services to meet the project requirements. The terms follow standard best practices with all costs itemized

and associated with project deliverables. Other than the benefits of having a successful project, there are no additional incentives (financial or otherwise) being offered to the researchers or health organizations for the use of the devices or software. There are no other obligations of the researchers towards the industry partners or any of the other partners or collaborators. The partners have no influence on the researchers' hypotheses/research questions, methods, analyses, results, or interpretation of these results. However, the clinicians, administrators, and patients and family caregivers will be invited to provide feedback on preliminary results emerging from the analyses.

3.2 Intellectual property.

The intellectual property pertaining to the research remains solely with the researchers. The intellectual property pertaining to the technology itself remains with the industry partners. Trinity Western University ("TWU" has an agreement with Cambian Business Services, Inc. ("Cambian") as the prime contractor. Collectively TWU and Cambian have properly managed the Intellectual Property and other contractual relationships following best practices.

3.3 Quality of Life and Practice Support System (QPSS)

The QPSS is an innovative, electronic health care information and practice support system for soliciting, assessing, and storing information provided by patients and family caregivers and making that information available to clinicians and decision makers to support person-centered care. The QPSS utilizes a flexible web-based platform that allows for the use of quality of life assessment questionnaires (including PROMs and PREMs) to solicit information from patients and family caregivers about their health, quality of life and care experiences. The QPSS has two Cambian-designed applications; one for patients and caregivers, the other for clinicians.

- *Cambian Navigator*: For patients and family caregivers to enter and review their information
- *Cambian Coordinator*: For clinicians to review patient and caregiver information as part of a quality of life assessment, using standardized reports, and to send requests to patients and caregivers about completing additional quality of life assessment questionnaires.

The QPSS can be accessed online using computerized devices (computers or tablets) with an internet connection, including mobile broadband or WiFi connectivity.

In addition to soliciting, assessing, and storing information, the QPSS facilitates:

a) the use of QOL assessment instruments at point of care via a computer or tablet to assess patients' and their family caregivers' QOL concerns and health care experiences (responses are either entered by the clinicians during assessment or independently by the patients and caregivers);
b) instantaneous feedback with information about scores, score interpretation[42], how scores have changed over time, comparison with established benchmarks, and targets for improvement;
c) person-centred care planning to address areas of unmet need; and
d) tracking and assessing whether an implemented intervention has achieved the desired result.

A key aspect of the QPSS is that it can be tailored to each particular practice setting by incorporating different assessment instruments that are appropriate for the patient/family population while addressing both clinical and administrative needs and adapting the feedback mechanisms based on user input. This Cambian applications have received privacy approval for use from Fraser Health, and meets standards for linkage with other health information systems.

Another key aspect of the QPSS is that user-centered design has been incorporated since its inception. Examples of features developed as a part of this process include: (a) an instrument session interface with a

focus on simplicity and usability with questions typically presented one at a time in large, clear text (see photo in Appendix 2), (b) an easy to use touch interface that takes into account differing levels of experience with tablets and varying levels of dexterity, (c) formatting of the questions to be consistent with the original paper-based instruments while taking advantage of the usability enhancements available on a tablet, (d) development of feedback/review interfaces that include both graphic and numerical reporting both at the level of individual questions and automatically calculated aggregate scores, and that support tracking and comparison of assessments over time.

4 JUSTIFICATION

The routine use of the QPSS has significant potential to enhance the delivery of care for people who have advancing life-limiting illnesses and who live at home. Rather than relying exclusively on the abilities of individual clinicians to assess patients' and family caregivers' perspectives of their needs in an *ad hoc* fashion, the QPSS ensures that the full range of QOL concerns and health care experiences are routinely assessed and made visible to clinicians in a systematic, interactive fashion. Similarly, the QPSS highlights the concerns and experiences of family caregivers at point of care. In addition, through integration with electronic health information systems, this patient- and family-centered information will become available for health services monitoring and quality improvement.

4.1 Expected Outcomes

The expected outcomes resulting from routine use of the QPSS are 1) provision of home care services for older adults who have a life-limiting illness that is more focused on patient and family caregiver QOL concerns, and 2) improved cost-effectiveness of the care provided.

First, we expect that the value of integrating the QPSS into routine home care will be shown in four distinct ways. 1) We anticipate that the health care system's ability to provide care centered on a comprehensive understanding of patients' and family caregivers' concerns on an ongoing basis will be enhanced. 2) There will be an increase in home health care teams' and administrators' abilities to monitor various QOL concerns of older adults and their family caregivers. The proposed interventions will facilitate clinicians' capacity to address patient and family caregiver concerns, and to the extent to which interventions are successful, they will further alleviate those concerns. 3) Patients' and family caregivers' satisfaction with the care provided to them will be increased when their needs and concerns are assessed and addressed. 4) Overall, QOL will improve for older adults and that of their family caregivers.

Second, through these mechanisms, we expect that integration of the QPSS into routine home care will increase cost-effectiveness. The administrative outcomes resulting from the integration of the QPSS with health information systems will enable cost-effectiveness analysis, program evaluation, and quality improvement initiatives informed by the concerns and experiences of both patients living with life-limiting illnesses, and their family caregivers. These outcomes will be achieved through early identification of QOL concerns so that potential crises that might otherwise result in emergency room or hospital admission can be prevented. Through the provision of supportive and effective care in the home, family caregivers' rates of burnout will also be reduced, thus reducing patient admissions to inpatient hospice, hospital, or long-term care. Cost-effectiveness will further be enhanced by enabling evaluation of different components of health services resulting in improved patient- and family-centered outcomes. Such appraisal includes health-economic evaluation based on health utilities derived from patient-reported outcome measures.

5 PURPOSE

This project seeks to address the above gaps by conducting outcomes evaluation research regarding the routine use of electronically administered QOL assessment instruments as practice support tools in the

home care of patients who have chronic life-limiting illnesses. Specifically, our proposed study is guided by four overarching aims and related research questions:

AIM 1: To examine the efficacy of the QPSS in home care for older adults who have advancing life-limiting illnesses.

- Does the routine use of the QPSS in home care improve quality of care, as indicated by patients' and family caregivers' reports of enhanced QOL and experiences with the care provided?

AIM 2: To evaluate the cost consequences of implementing the QPSS in home care.

AIM 3: To understand the process of implementing the QPSS in home care.

- How can we best facilitate the integration and routine use of the QPSS in the home care for older adults who have advancing life-limiting illnesses and their family caregivers?

AIM 4: To examine how integration of the QPSS with health information systems will meet the needs of administrators for quality improvement and accreditation.

For the first aim, we will conduct a randomised controlled trial (RCT) to evaluate the impact of using the QPSS on QOL and health care experiences of patients and their family caregivers by testing the following **hypotheses**: Use of the QPSS in routine home care clinical practice will result in improved QOL [primary outcome] for older adults who have life-limiting illnesses [Hypothesis 1] and for their family caregivers [Hypothesis 2], as well as increased patient [Hypothesis 3] and family caregiver [Hypothesis 4] satisfaction with care [secondary outcome] compared with these measures in patients and caregivers who receive usual home care.

For the second aim, we will evaluate the impact of implementing the QPSS on resource use and costs (health care *and* non-health care costs) from a cost-consequences to society perspective (consequence analysis framework), which will be complemented by a cost-utility analysis.

For the third aim, we will examine how the QPSS is being used in practice. Specifically, we will use mixed methods to (a) understand clinicians', patients', and family caregivers' points of view about how to adapt and integrate the QPSS intervention into clinical practice at each home care office; (b) examine how the QPSS is used to support care planning locally by linking assessment data to procedures adopted at each home care office and tracking interventions and practices of the interdisciplinary team to address patient and caregiver QOL concerns, and (c) evaluate the process of using the QPSS in home health care.

For the fourth aim, we will examine how the QPSS can be integrated broadly with other health information systems to meet the needs of administrators at various levels (home care office; health authority; Ministry of Health) and enable patient- and family-centered analytics for continuous quality improvement and accreditation. Actual integration will not occur during the project.

6 METHODS

6.1 Overview of Research Design

The original REB application involved only the focus groups. This amendment pertains to the randomized control trial (RCT) and process evaluation in Stage 2 of the project. The research design for all three stages is briefly described so that it is clear how each stage relates to the overall project. The economic evaluation component of Stage 2 and the sustainability study in Stage 3 will be detailed in a future amendment for research ethics approval.

This project is for the evaluation of both the process of implementing the QPSS into home care practice[57-60] and the outcomes resulting from it. The use of the QPSS is a complex intervention because it has several interrelated components, presents several challenges for evaluators, and has methodological and practical complications that must be addressed.[59] Adaptation of the intervention to local context and engagement of local health care teams is critical for successful implementation. We will take an action-oriented approach by applying the CIHR Knowledge to Action (KTA) Framework[61, 62] in three stages (see Appendix 3), and involve clinicians, administrators, patients, and family caregivers in the adaptation and integration of the QPSS into routine practice. We will assess facilitators and barriers to the use of the QPSS,[51] find ways of overcoming barriers, implement the QPSS in routine care, evaluate outcomes, and sustain knowledge use.

6.1.1 Stages of the research project

Stage 1: Local adaptation (one year at each home care office).

- Pre-intervention measurement of QOL and health care experience outcomes (prior to use of the QPSS).
- Iterative qualitative data collection and analysis to guide adaptation and integration of the QPSS intervention in local settings (home care offices), which will also inform **Aim 3**.
- Development of site-specific protocols for QPSS integration into routine daily practice. These protocols will be iteratively adapted to changing healthcare delivery systems over the duration of Stage 1.
- Once the QPSS is adapted for local use and a protocol for integration into practice elaborated, clinicians will receive education about the QPSS and how to integrate it into their practice, and then integrate it into practice in the last 6 months of Year 1 at their site as a period of transition. Data of the patients and family caregiver who participate in the transition phase will not be collected as part of outcome evaluation.

Stage 2: Evaluation (one year at each home care office). Focusing on the KTA phases of monitoring knowledge use and evaluating outcomes, this stage will involve:

- Conducting a RCT to evaluate the impact using the QPSS on the QOL and health care experiences of patients and family caregivers **(Aim 1)**.
- Conducting an economic evaluation to estimate the cost consequences (and cost-utility) of implementing the QPSS in home care **(Aim 2)**.
- Applying qualitative and quantitative methods to examine the process of QPSS use in home care **(Aim 3)**.

Stage 3: Sustainability (from the end of Stage 2, which depends on the timing of enrollment of each home care office to six months prior to the end of the grant)**.** This stage will be guided by the last part of the KTA framework aimed at local sustainability and dissemination of lessons learned. Specifically, Stage 3 will include:

- Examining the integration of the QPSS with other health information systems and use of QPSS data by health care administrators **(Aim 4)**.
- Discussion of results of all parts of the evaluation among the team members regarding whether sustained and more widespread integration into practice is warranted.
- If the results warrant, discussion among team members as to how to scale up the intervention.
- Broad dissemination of the results and lessons learned.

6.1.2 Experimental design

To test the hypotheses for Aim 1, we will conduct a two-arm parallel RCT with treatment and control groups (Stage 2). Patients and their family caregivers recruited in Stage 2 will be randomly assigned to a treatment group (clinicians use the QPSS and have access to QPSS data) or control group (clinicians do not use the QPSS and will not have access to the QPSS data), stratified by site. Stratification is necessary because the intervention protocol may not be identical for the different home care offices. To explore the possibility of contamination (since clinicians will be caring for patients and caregivers in both arms) and the potential impact of changes in the health care system between Stages 1 and 2, we will also collect outcomes data collection from a pre-intervention group consisting of QPSS-naïve patients and family caregivers enrolled during the 12 months of Stage 1 at each site, before the QPSS is used in practice.

6.2 Methods for Stage 1: Local integration and exploratory use of the QPSS intervention

Qualitative methods, including focus groups with clinicians and managers, as well as interviews with patients and family caregivers at each site, will be used with the goals of tailoring the QPSS intervention to each local practice setting and developing site-specific implementation protocols. In Stage 1, at each home care office we will: (a) determine the selection of appropriate QOL assessment instruments to be implemented in clinical practice using the QPSS; (b) address barriers and identify facilitators for workflow integration; (c) work with the health care teams to identify site-specific practices and interventions to address concerns that may be identified by the patients and family caregivers in Stage 2 (the range of concerns that may be identified will be determined by the locally selected QOL assessment instruments); and (d) finalize an implementation protocol for integration of the QPSS into routine practice. During the last six months of Stage 1, we will have the clinicians at each home care office explore the use of the QPSS intervention in practice (transition period) (see Appendix 4).

6.2.1 Development of the QPSS implementation protocol

A selection of validated QOL assessment instruments (see Appendices 5 and 6) has been programmed into the QPSS based on findings from our preliminary studies, both of which were informed by our knowledge synthesis on Patient- and Family-Reported Experience and Outcome Measures for Elderly Acute Care Patients.[63] It is neither realistic nor desirable for all of these instruments to be used in each home care office. The selection of instruments must be informed by site-specific considerations, including the composition of the home care team and team-members' prior familiarity with any of the QOL instruments. Therefore, based on the results of the Stage 1 focus groups, we will collaboratively determine which QOL instruments are most suitable as practice support tools. To ensure that the needs of both patients and family caregivers are considered, each home care office will be asked to select at least one instrument for patients and another for family caregivers.

Protocols from home care offices enrolled earlier on in the project will be used to inform the protocols of subsequently enrolled sites. In addition to the selection of instruments, the QPSS intervention will be locally adapted (e.g., linkages of assessment results with site-specific practice guidelines and procedures, and strategies for using the assessment information for interdisciplinary care planning and shared decision making). To control for and examine this variability between sites, different site-specific characteristics of the intervention protocol will be included as moderator variables in the analysis.

The QPSS site-specific implementation protocol will be finalized during Stage 1 at each site.

6.2.2 Data collection from the pre-intervention comparator group

During this time before the QPSS is used in practice, a research staff will recruit patients and family caregivers and collect outcomes data for the pre-intervention comparator group. Data on pre-intervention measurement of QOL and health care experience outcomes (prior to use of the QPSS) will be collected (by

tablet, phone, mail or internet). This data will be used to test the recruitment and data collection procedures and explore the possibility of contamination and the potential impact of changes in the health care system between Stages 1 and 2.

6.2.3 Transition phase to explore use of the QPSS intervention

During the last six months of Stage 1, a few selected clinicians will be invited to volunteer use of the QPSS. The clinicians will be asked to provide feedback on the use of the QPSS in their practice via the focus groups and in interviews, with the goal to inform the development of a site-specific protocol for its implementation in Stage 2. In this transition phase, the patients and family caregivers who have used the QPSS will be invited to participate in an interview to share their experience in using the QPSS. The feedback obtained from the patients and family caregivers interviewed in this phase will also inform the development of the QPSS implementation protocol at each site.

6.3 Methods for Stage 2: Evaluation of outcomes, cost-effectiveness and processes of use

The evaluation stage will include evaluation of patient- and family-caregiver outcomes, cost-effectiveness, and the processes of using the QPSS. This stage consists of testing the four hypotheses of the impact of using the QPSS intervention on patients' and caregivers' outcomes, including their QOL and experiences with the care provided. Following a parallel groups RCT design, patients and their family caregivers will be randomly allocated to an intervention group or a usual care group within each of the home care offices. The QPSS implementation protocol will be followed with all patients and family caregivers from the time of their enrollment in Stage 2 for a period of one year (see Appendix 4). This study has been registered with ClinicalTrials.gov (NCT02940951), and updated prior to trial commencement.

6.3.1 The QPSS intervention.

As described in the Introduction and Background sections, the QPSS intervention involves making the QOL information provided by patients and family caregivers available to clinicians and decision makers, to support person-centered care. The implementation of the QPSS will follow the protocol developed with clinicians at each site during Stage 1. Once every two months, patient and family caregiver participants will complete the set of questionnaires aligned with their respective roles via the web application (Cambian Navigator). Patients will complete: MQOL-E[64, 65], CANHELP LITE (patient) [66], VR-12[67] and ESAS-R[68, 69]. Family caregivers will complete: QOLLTI-Fv2[70], CANHELP LITE (family caregiver) [66], VR-12[67] and CSNAT [71, 72]. The list of questionnaires is in Appendix 18; the questionnaires are described in Appendices 5 and 6 and in the latter section on outcome measures in the study protocol.

Clinicians will have access to participants' responses to the following QOL questionnaires completed by the participants in the intervention group via the clinician's individual Cambian Coordinator account:

- Patient: MQOL-E, ESAS-R
- Family caregiver: QOLLTI-Fv2, CSNAT

Clinicians will review the information and determine the appropriate follow-up actions, as part of their care planning for the intervention group participants. A schematic depiction of the QPSS intervention is in Appendix 31. Clinician's will not have access to participants' to the VR-12 and CANHELP LITE (patient and family caregiver versions). These questionnaires are included for purposes of outcomes evaluation, cost-consequence analysis (VR-12), and quality improvement. However, data on these questionnaires will be made available to Fraser Health decision makers (management and administration) for purposes of quality improvement, reporting, and accreditation.

6.3.2 Intervention group

In the intervention group, patients and family caregivers will use the Cambian Navigator system. Clinicians *will be asked to* use the Cambian Coordinator system, and will have access to some of the data collected

from the patients and family caregivers in this group. The site-dependent QPSS implementation protocol for follow up (developed during Stage 1 at each home health site) will be applied to all patients and family caregivers in the intervention group.

6.3.3 Control group

In the control group, patients and family caregivers will use the Navigator system independently of the clinicians. Clinicians *will not* use the Coordinator system with this group of patients, nor will the data collected from the control group be made available to clinicians. Clinicians will not know the identity of the patients and family caregivers in the control group, and this group of patients and family caregivers will continue to receive care as usual from their clinicians.

6.3.4 Allocation to intervention and control conditions

Consenting patients at each home care office will be randomly assigned on a 1-to-1 basis to the intervention or usual care group using a computerized random number generator. Randomization will be stratified by site. To ensure that both members of patient-family caregiver dyad are assigned to the same group, consenting patients will be randomized (whether or not they intend to provide outcome data) and if they have a participating family caregiver, the caregiver will be assigned to the same group. Dr. Robin Cohen, co-PI, is in Montreal and completely removed from the recruitment and allocation process. She will generate and download the randomization sequence, place group allocation on a folded paper inside sequentially numbered opaque envelopes for each site, and mail all envelopes to Dr. Sawatzky. She will store a copy of the randomization sequence in a locked filing cabinet in a locked office in Montreal.

The research assistant will inform the research coordinator as soon as a patient (and family caregiver if applicable) is recruited. The coordinator will open the next sequentially numbered allocation envelope to reveal group assignment. She will immediately inform the clinicians in the respective home care office of any patients/family caregivers allocated to the intervention group via generic email notification.

6.3.5 Concealment of group allocation

Only the research coordinator and clinicians will know which patients and family caregivers are assigned to the intervention group. Patients and family caregivers will not be informed as to group assignment, although those in the intervention may learn of it from the clinician. Instead, all patients and family caregivers will be told that their home health care providers may or may not have seen their completed questionnaires. This is intended to reduce the risk that patients and family caregivers assume that their health care providers are aware of any problems highlighted in their questionnaire responses. This is important for both groups, since we do not know the extent to which clinicians will actually access the completed questionnaire scores of those in the intervention group. Research assistants collecting data will be blinded as to group allocation. To prevent unblinding of the research assistants during data collection, they will ask patients and caregivers not to talk to them about their home health providers' use/not use of their questionnaire scores. The research assistants collecting the data will be blinded to treatment or control group allocation.

6.3.6 Contamination

Clinicians who perceive a positive impact on their practice through awareness of the QOL scores of their patients and family caregivers in the intervention group may change their practice to more comprehensively assess QOL for their other patients, some of which may be in the control group. Patients and family caregivers in the control group may choose to discuss their QOL more with their health care providers, or even print and share their completed QOL questionnaires. These are desirable outcomes, but in an RCT, it is considered contamination. Therefore, no effort will be made to either encourage or prevent this, but we will assess such contamination in two ways. First, as stated above, the QOL and experience

with health care scores of patients and family caregivers from the pre-intervention group (collected in Stage 1) will be compared to the scores of the control group (Stage 2). Second, clinicians participating in focus groups and individual interviews will be asked to describe any change they've noticed in their general practice related to their experience with the intervention group (see revised clinicians focus group guide in Appendix 16 and interview guide in Appendix 29).

6.3.7 Assessing adherence

The QPSS will capture information about the frequency and timing of the completion of QOL questionnaires by patients and family caregivers, as well as the viewing of scores by clinicians. In addition, in interviews with all types of participants, we will explore the ways they used or did not use the QPSS and their reasons for doing so (see revised interview guides in Appendix 29 and 30).

6.3.8 Quantitative data collection (questionnaires)

All patient participants and family caregiver participants providing outcomes data will complete all the questionnaires at baseline and every two months for a period of one year. Patients will complete: MQOL-E[64, 65], CANHELP LITE (patient) [66], VR-12[67] and ESAS-R[68, 69]. Family caregivers will complete: QOLLTI-Fv2[70], CANHELP LITE (family caregiver) [66], VR-12[67] and CSNAT [71, 72]. The list of questionnaires is in Appendix 18. The Cambian Navigator system will also record detailed usage metrics (frequency and timing of different assessment instruments, time to complete the assessments, viewing of reports).

At baseline, questionnaires will be administered by a research assistant during an in-person meeting at the patient's or family caregiver's home, or via a phone meeting (depending on the participant's preference). Participants have the option of using their own devices, or a project-tablet provided by the researcher assistant during a home visit to complete the questionnaires using the Cambian Navigator application. The questionnaires could be completed independently by the participant or via an interview with the research assistant. If preferred, participants may also choose to return the questionnaires by mail after the meeting. A stamped envelope will be provided. In this case, and in situations where the application is not accessible (e.g., due to poor mobile broadband connectivity), questionnaires will be completed on paper and entered into the Cambian Navigator application on the participant's behalf later on.

Participants will be asked to select any of the following options for completing follow up questionnaires (every two months from start of enrollment for 12 months or until discharge from home care services or death):

1) by using their own computer or tablet to access the Cambian Navigator application and complete questionnaire independently,
2) with a research staff member via an in-person home visit following the same procedures as above for the baseline questionnaire,
3) by having the research staff member administer the questionnaires over the phone,
4) by completing the questionnaire independently on paper (to be returned by mail).

6.3.9 Data collection from health records

The cost-consequences evaluation will run in parallel with other components (see "Analysis of administrative data in cost-effectiveness evaluation" for further description).

6.3.10 Qualitative data collection (focus groups and interviews)

In Stage 2, clinicians will only use the QPSS with participating patients and family caregivers in the intervention group as part of their practice. We will explore their experiences of using the QPSS by inviting clinicians to participate in focus groups and individual interviews. The interviews allow for more in depth responses that are not influenced or mediated by others in the focus groups. We will also conduct one-

time interviews with patients and family caregivers in the intervention group to understand their perspectives regarding use of the QPSS and we will bring this coded information to the focus group discussions. As explained in the above section on assessing contamination, clinicians participating in focus groups and individual interviews will also be asked to describe any change they've noticed in their general practice related to their experience with the intervention group.

6.4 Sampling Design and Subject Selection

6.4.1 Clinicians

6.4.1.1 Inclusion criteria
Clinicians who are working with participating home care offices and who are providing care to older adults with life-limiting illness; clinician participants may include nurses, nurse practitioners, physicians, physiotherapists, occupational therapists, social workers.

6.4.1.2 Exclusion criteria
Clinicians who are neither providing home care for older adults with life-limiting illnesses nor working with a participating home care office.

6.4.1.3 Stage 1
Clinicians from various disciplines will participate in the focus groups. These clinicians will be recruited from 7 home care offices (units) in 2 health authorities (Fraser Health and Alberta Health Services) in 2 provinces (BC, Alberta). The number of clinicians participating in the study will average between 4 and 8 per unit; some sites may have more than 8 clinicians participating in some of the focus groups, pending on the site size and clinicians availability to attend the focus group. The home care offices were enrolled during the first year of the project.

6.4.1.4 Transition phase
For the transition phase during Stage 1, 2 to 4 clinicians participating in the focus groups at each home health offices will explore use of the QPSS with 1 to 2 of their patient(s) and family caregiver(s) of their choice who are not providing pre-intervention outcomes evaluation data. Clinicians who have used the QPSS in the transition phase of Stage 1 will be interviewed about their experience in using the QPSS, in addition to providing feedback at the focus groups. All home care offices are currently enrolled in Stage 1 of the project.

6.4.1.5 Stage 2
Each home health office will transition into Stage 2 of the project between November 1 2017 and October 30 2018. The exact date for transitioning to Stage 2 will be determined with management at each site in accordance with the timing of Stage 1. See anticipated schedule in Appendix 4. Similar to Stage 1, clinicians at the 7 home health sites enrolled in the project will be invited to participate in Stage 2 focus groups. Between 4 and 8 clinicians per site will be recruited for the focus groups. A purposive sample of clinicians who have used the QPSS will be individually interviewed. They will be sampled for profession and frequent versus rare users. We anticipate between 18 and 24 interviews, depending on when data saturation is reached. Clinicians who have used the QPSS will also provide feedback at the focus groups.

6.4.2 Patients and family caregivers

6.4.2.1 Inclusion criteria
1. Patients 55 years of age and older who are receiving ongoing home care due to advancing life-limiting illnesses.

2. Family caregivers are identified as eligible and competent by the patient, or by a clinician if the patient is unable to do so, and as the person most involved in the care of a patient who meets the eligibility criteria just above (#2). No more than one caregiver per patient will be recruited.
3. Patients and family caregivers have the ability to speak English and capacity to provide informed consent.

6.4.2.2 Exclusion criteria

Patients who are less than 55 years old and are not identified as having one or more advancing life-limiting illnesses.

6.4.2.3 Stage 1

For the outcomes evaluation (Aim 1), a pre-invention comparator group totaling 180 patients and 180 family caregivers, will be recruited (see Appendix 4). The number of participants per site will vary depending on the size of each site.

6.4.2.4 Transition phase

Selected clinicians participating in the focus groups at each home health offices will explore the use of the QPSS with 1 to 2 patient(s) and family caregiver(s) at each site; these patients and family caregivers are not at the time providing pre-intervention outcomes evaluation data. Clinicians will select these patients and family caregivers using convenience sampling. These patients and family members will also be asked to participate in a one-time interview about their experiences of using the QPSS.

6.4.2.5 Stage 2

For the RCT, we will recruit a total of 360 patients and 360 family caregivers across all sites (see Appendix 4). The number of participants at each site will vary depending on the size of the site. The RCT participants may include Stage 1 participants, but will not include individuals who have used the QPSS in the transition phase; they may choose to continue using the QPSS if they wish but any data they provide will not be used in the study. Eighteen to twenty-four of the RCT participants will be interviewed about their experiences using the QPSS during this stage. The interviewees will be purposively sampled from the intervention arm, to achieve diversity in gender, age, health condition, and family caregivers' relationship (e.g., spouse, child, or friend).

6.5 Recruitment and Informed Consent

6.5.1 Clinicians

6.5.1.1 Stages 1 and 2.

All members of the home care team, except the Edmonton Zone, will be informed about the study via a letter of invitation from the study team (Appendices 8 and 8a for Stage 1; Appendix 36 for Stage 2) that will be distributed via email by the manager or designated person at the Fraser Health home health offices. The consent form will be included with the email (Appendices 9 and 9a for Stage 1; Appendix 37 for Stage 2). For the Edmonton Zone, the Alberta Health Services Continuing Care Edmonton Zone Home Living Program will be the study site. All members of this home care team will be informed about the study via a letter of invitation from the study team. The letter of invitation will be distributed via email by the site champion, who is also one of the study collaborators, at the home care office. The Alberta Health Services (AHS) Research Ethics Board has provided approval to commence Stage 1 (PRJ #34495). See Appendix 38 for approval certificate from AHS.

Where possible, a research team member and a research staff member will attend a team meeting with clinicians at the home care offices to explain the study and answer questions. Clinicians who are interested

in participating will indicate their interest at this meeting, or by contacting the research team member or the research staff member within a week after the meeting. Clinicians who are unable to attend the in-person meeting will be invited to telephone or email the research staff in response to the invitation letter. The research staff will then explain the study and consent form over phone or email. For the Edmonton Zone, clinicians who are interested in participating will also write down their contact information on a sign-up sheet at this meeting. Written informed consent will be obtained from the clinician participants at the first focus group, after the facilitators of the focus group have explained the consent form to the potential participants. Written consent will be obtained prior to interviews, after the research personnel has explained the consent form. Clinician interview consent form used in Stage 2 will be similar to the consent form approved for Stage 1 use (Appendix 28).

6.5.2 Patients and family caregivers

6.5.2.1 Stage 1

For all home care services sites, except the Edmonton Zone, an employee from Fraser Health will screen for eligible patients and family caregivers. The employee will have pre-existing authorization to access patient records and will screen patients and family caregivers using the inclusion and exclusion criteria described above. Potential family caregivers will be identified by the patient as the family member most involved in the patient's care. No individual patient information from the screening process will be shared with the research team. The employee conducting the screen will provide the research team only with aggregate information about reasons for inclusions and exclusions resulting from the screening process.

For the Edmonton Zone, at the initial clinician focus group, clinicians will be asked to invite clients and caregivers to obtain consent for researchers to contact them about the study. Clinicians will read the consent to contact script and will give clients and caregivers the client study information letter and the consent to contact form to complete. Clinicians will invite clients and caregivers who they would normally interact with in their usual daily practice, and who also meet the inclusion criteria. The signed consent to contact forms will be put in an envelope marked with the study name and stored in a locked filing cabinet in the Edmonton Home Care Office. Once research team members obtain these forms, they will then contact (via telephone or email) home care patients and caregivers to invite them to take part in the pre-intervention outcome surveys.

Patients and family caregivers will be enrolled in the study, using the approach for recruitment and informed consent mapped to the respective scenario: 1) with prior consent to contact for research; 2) without prior consent to contact for research; 3) clinician referrals.

Scenario 1: With prior consent to contact for research
The health authority employee will first identify the panel for potential participants and cross-reference patients who have already provided Consent to Contact for Research Purposes as documented in Meditech. If prior consent to contact has been obtained, the FH staff will call the eligible patients to confirm their agreement to be contacted for research and ask if they are interested in learning more about the study. If they indicate an interest in the study, the health authority employee will do the following:

1. Determine consent for the research team to contact the patient and confirm the patient's contact information (mailing address, phone, and email).
2. Ask the patient to identify the family caregiver who is most involved in his/her care.
3. Mail the following recruitment materials to patients and family caregivers who agree:
    i) Family caregiver consent to contact form (Appendix 10)
    ii) Study introductory letter (Appendix 11a)
    iii) Patient and family caregiver letter of invitations for participation in the study (Appendices

12 and 13)

iv) Patient and family caregiver consent forms (Appendices 14 and 15)

v) Stamped self-addressed envelopes will be included with the above material.

4. If desired, set up an appointment for a research personnel to follow up by phone or conduct an in-person visit scheduled for a minimum of a week after the initial phone contact to review project information, answer any questions, and obtain written consent to participate. As described in the Privacy Impact Assessment, once consent has been obtained, research staff will help clients who are participating in this stage and caregivers register on the Cambian Navigator system with an email address and a unique password and to use the Navigator system and if desired, assist with using the Navigator system.

Scenario 2: Without prior consent to contact for research
If no prior consent has been obtained, the Fraser Health employee, on behalf of Lisa Zetes-Zanatta (Fraser Health Executive Director) will send a letter with introductory information about the initiative (Appendix 11b) and the consent to contact forms to the patients and their family caregivers (Appendix 10). Stamped self-addressed envelopes will be provided for individuals who consent to be contacted to return their signed consent forms. After receiving their written consents to contact, the recruitment and informed consent process will be identical to the steps in recruiting and ascertaining informed consent scenario 1 described above, starting at Step 2.

For individuals who wish to be contacted by mail only, the study recruitment package will be mailed to them after receiving consent to contact. If the patients and family caregivers contacted the research team to provide verbal consent to contact and to receive information about the study, the research team will then mail the study recruitment package to the consented individuals.

Scenario 3: Clinician referral
During routine clinical communication, clinicians may also ask patients and family members if they are interested in being contacted for the study. Clinicians, except those in the Edmonton Zone, will obtain the contact information of those who have agreed to be contacted and convey the information to the research team via phone. In the Edmonton Zone, clinicians will put the consent to contact forms in an envelope and store it in their site. A research personnel will pick up the envelope. Research personnel will follow up with a phone call to the potential participants to ask if they are interested in learning more about the study. If they indicate an interest in the study, the recruitment and informed consent procedure will be identical to scenario 1 described above, starting at Step 2.

In all the above scenarios, if the potential participants cannot be contacted at the time of the call, research personnel will use the following script to leave a voice message, where possible:

“Hello Mr/Mrs/Ms, it’s (caller name) calling from Trinity Western University/University of Alberta. I’m following up with the information you’ve received regarding the study on integrating a quality of life assessment and practice support system in homecare services for older adults with chronic illness and their families. It’s (date and time). I will call you again tomorrow morning. You can also call me anytime tomorrow at the call-back number (1-877- 898-4630 for TWU). Thank you very much and I look forward to speaking with you.”

6.5.2.2 Transition phase

The initial contact with potential participants will be made by a clinician who is: 1) responsible for providing care in the home, and 2) participating in exploring use of the QPSS. Patients and family

caregivers who are eligible (see inclusion criteria above on page 21) will be invited to use the QPSS. The clinician responsible for patient care will confirm eligibility of the patients and family caregivers. Eligible patients and family caregivers include those who meet the inclusion criteria and are not part of pre-intervention comparator group or who are part of the pre-intervention group but have already completed responding to the four administrations of the pre-intervention questionnaire (i.e., after recruitment for the pre-intervention group has been completed). The clinician will explain the study using the following script:

"In Fraser Health homecare/Alberta Health Services, we are currently conducting a research project to understand if and how we can improve our care to you by using an electronic system called the QPSS. We would like to invite people to try using the system. If you are interested and permit the research team to contact you for this project, I will give the research team your phone number."

For those who agree to be contacted, clinicians, except those in the Edmonton Zone, will obtain the contact information of those who have agreed to be contacted and convey the information to the research team via phone. In the Edmonton Zone, clinicians will put the consent to contact forms in an envelope and store it in their site. A research personnel will pick up the envelope. Research personnel will follow up with a phone call to the potential participants to ask if they are interested in learning more about the study. If they indicate an interest in the study, the recruitment and informed consent procedure will be identical to the steps described above for those who have already provided consent to contact for research purposes (see Patient QPSS consent form in Appendix 23 and Family caregiver QPSS consent form in Appendix 24).

When patients and family caregivers agree to participate in the transition phase of the study, they will be advised that they are also be invited for an interview regarding their experiences with the assessment instruments and the electronic system. Participants will be advised that they may refuse to participate in the interview or withdraw at any time. Consenting patients and family caregivers will be asked if they are willing to participate in a subsequent interview about their experience of using the electronic system. Consent for interview will be achieved through a separate consent process after using the electronic system (Appendices 25 and 26). Interviews will be conducted by a research personnel during the same visit as the system use, or in a follow-up separate visit, as per the participant's preference. The interview may take up to 60 minutes.

6.5.2.3 Stage 2

Participants who are already part of Stage 1 will be invited to participate in Stage 2, with the exception of those who participated in the transition phase. Additional participants will be recruited following the same procedures as described above in Stage 1. However, the letter of invitation and consent forms used in Stage 1 will be replaced by the following appendices to be used in recruitment and informed consent in Stage 2:

i) Patient and family caregiver letter of invitations for participation in the study (Appendices 32 and 33)
ii) Patient and family caregiver consent forms (Appendices 34 and 35)

A purposive sample of the patients and family caregivers in the intervention group will be invited to participate in a one-time interview regarding their experiences with the assessment instruments and the electronic system. Consent from individuals who agreed to be interviewed will be sought through a separate consenting process. Consent forms for interviewing patients and family caregivers in Stage 2 are similar to those provided in Appendices 25 and 26.

6.6 Data Collection

6.6.1 Clinician focus groups and interviews

The demographic information form (see Appendix 17) will be administered during the first session of the focus groups, and for any new participants in their first session. Clinicians who have used the QPSS in Stages 1 and 2 will be asked to provide feedback on their experience by participating in focus groups. The clinicians who have experiences of using the QPSS will also be invited to join a one-time individual interview, either in person or via phone, other than the focus groups. Questions to guide the interviews with clinicians are provided in Appendix 29. For any clinician who has not provided demographic information in the focus groups, this information will be collected from them before the beginning of the individual interview. Focus group sessions and interviews may last up to 90 and 60 minutes, respectively. All focus groups and interviews will be audio-recorded and transcribed.

To assess the cost of local adaptation of the QPSS implementation protocol and training for its use, the research team will track the time participating staff spent on Stage 1 activities as reflected by the scheduled meetings (focus groups and trailing training), and also through interviews with clinicians who have used the QPSS.

6.6.1.1 Stage 1 and transition phase

Clinician participants will be asked to participate in focus groups once every two months in Stage 1 at each home care office (a total of 4-5 sessions per participant). Questions to guide the focus groups are provided in Appendix 16. Clinicians who have used the QPSS with patients and family caregivers in Stage 1 transition phase will also be asked to provide feedback on their experience during the focus groups and in one time in-person interviews.

6.6.1.2 Stage 2

Clinician participants who are using the QPSS with patients and family caregivers will be asked to participate in 3 to 4 focus groups at each home care office. Questions to guide the focus groups will be drawn from the focus group guide used in Stage 1 (see revised Appendix 16). A purposive sample of clinicians who have used the QPSS will also participate in a one-time interview of about 60 minutes in duration.

6.6.2 Patient and family caregiver outcome evaluation data

6.6.2.1 Stages 1 and 2

Patients and family caregivers participating in the study will be asked to provide basic demographic and health information prior to completing the QOL questionnaires. Demographic questionnaire for both stages is found in Appendix 18. At each stage, QOL and health care experience data of patients and family caregivers will be collected once every two months in the 12 months of Stage 1 (pre-intervention outcome group) and over a period of 12 months for Stage 2 (both control and intervention groups) or until discharge from home care services or death by all patients and family caregivers in both stages. Prior to randomization (Aim 1 of Stage 2), baseline data collection will be completed. Participants may complete the questionnaires independently on paper (and returned via mail) or online (using a web-enabled computerized device and the Cambian Navigator system), or with support during a follow up phone call or home visit by the research personnel, as described above. Subsequent data collection questionnaires will be administered every two months via the modality (paper, online, home visit, or phone) of participant preference. The time required for patients and family caregivers to complete the questionnaires will be approximately 30 minutes for most participants, as informed by earlier catalyst QPSS projects (see Appendix 18 for QOL questionnaires).

6.6.2.2 How patients and family caregivers may choose to use the Navigator system

Following Dillman's guidelines for mixed-mode surveys,[73] participants will have the option to complete the questionnaires independently using their own web-enabled devices, with a research staff via an in-person home visit, by phone, or by mail, to be determined during the initial contact for obtain informed consent, as described above. The following data collection options encompass Stage 1, and both intervention and control arms of the Stage 2 RCT.

- Using their own computer or tablet to access the Cambian Navigator application and complete questionnaire independently

Participants who chose to complete the questionnaires independently using their own web-enabled device will be sent email reminders from the research team every two months. Participants will then log into their respective Cambian Navigator account to complete the online questionnaires. The research staff will provide the participants an instruction sheet to guide their subsequent use of the Navigator system to complete their questionnaires online (see Appendix 27).

- Completing the questionnaire with a research staff member via an in-person home visit

After the initial in-person home visit enrolment and baseline data collection, participants may choose to continue completing questionnaires with a research staff member through home visits. The questionnaires will be administered as a face-to-face interview with the research staff, using their own web-enabled devices or a SIM card enabled project tablet. The research staff will similarly provide the participants with the instruction sheet for their use of the Navigator system (see Appendix 27). In instances where internet connectivity is limited, paper questionnaires will be used and the information will subsequently be entered into the participant's respective account set up in the Cambian Navigator by the research staff.

- Having the research staff member administer the questionnaires over the phone

For participants who chose to complete the questionnaires by phone, the research personnel will call the participant and administer the questionnaire by reading out each question and eliciting their response. If the participant is not available at the time of the phone call, a voicemail will be left and the research personnel will follow up with another phone call within the next two days. The information will be entered into the Cambian Navigator on behalf of the participants. All participants will be regularly contacted by a research personnel to complete the questionnaires in accordance with the data collection schedule.

- Completing the questionnaire independently on paper (to be returned by mail)

For participants who chose to complete the questionnaires by mail, a paper copy of questionnaires will be mailed every two months with instructions (see Appendix 20) and a stamped and self-addressed envelope. The instructions will include the phone number for contact should there be any questions. Participants will be instructed to not include their return address on the stamped envelope. The research staff will enter the information on the paper-based questionnaires in the participant's respective Cambian Navigator account.

6.6.3 Patient and family caregiver interview data

Consenting patients and family caregivers will be asked to participate in one interview about their experience using the QPSS in the transition phase and Stage 2. Interviews will be conducted by a research personnel, and interviews may take up to 60 minutes. Each interview will be conducted in person at a time convenient to the participants. Questions to guide the interviews with patients and family caregivers are provided in Appendix 30. Interviews and will be digitally recorded and subsequently transcribed. All identifying information will be removed during transcription. The research team will have access to de-identified transcripts.

6.6.4 Patient and family caregiver administrative data

In addition, participants' healthcare administrative data will be requested from healthcare provider records and Population Data BC (www.popdata.bc.ca) for Fraser Health and SPOR Platform for Alberta Health Services. This will include information about health services utilization and medical history, which will be used for a cost-consequences analysis. To enable linkage with administrative data sources, each participant is requested to provide their personal health number, as is indicated on the consent form. All information collected from these records will be kept in a separate database after personal identifiers have been removed. We will link the information collected from these records to the participant survey information. Only the PI and research personnel will have access to the identifier that links participants' survey information to their medical records and the administrative data. A data access request will be submitted to obtain access to this information prior to proceeding with the cost-consequences analysis.

6.6.5 Data regarding use of the Cambian applications

In addition to the questionnaire data, the Cambian applications will record data about how long it has taken for patients and family caregivers to complete each questionnaire, how often and how long each clinician has interacted with the system, which qualitative of life information has been reviewed by the clinicians. The de-identified usage data will be available to the research team to evaluate implementation of the intervention and as part of the economic cost-consequence analysis.

6.7 Use of the QPSS in practice during the transition phase and Stage 2

Research personnel will show the participant (patient and/or family caregiver) how to use the Cambian Navigator system via a tablet during the initial in-person visit. The participant will decide whether or not to use the tablet by themselves or to have it administered as a face-to-face interview. Similarly, participants opting for independent use of the Cambian Navigator will be provided the instruction sheet (see Appendix 27). If a person is not able to use the tablet on their own, the research personnel will be able to elicit responses by interviewing the patient using the questions and noting responses on the tablet themselves. The questionnaires (assessment instruments included in the QPSS) consist of questions with fixed response options (e.g. rating scales). There are a few questions in which people can enter a short descriptor of no more than few words in length (e.g. a symptom). Participants will be asked to complete the questionnaires using the QPSS, either independently or with a research personnel's help, before clinicians' visit.

As detailed in the Privacy Impact Assessment, each clinician using the QPSS is enrolled as a separate user on Cambian Coordinator and signs-in using a personalized login (username and password). Once signed in, the clinician has the appropriate level of access to find participants and review previously recorded information for follow up. Clinicians will have access only to the QPSS data of patients and family members enrolled in the transition phase and in the intervention arm of the RCT in Stage 2 from their respective Coordinator account. Clinicians will use Cambian Coordinator to access this data. The research coordinator will notify the clinicians at the respective sites via a general email (without participant identifiable information) to the clinician point-person identified by each site when an outcome evaluation assessment has been completed (see Appendix 39 for Coordinator instruction sheet for clinicians).

In the transition phase and intervention group of Stage 2, clinicians will use their clinical judgement to decide whether they will invite the patients and family caregivers to complete particular questionnaires more than once by sending invitations via Cambian Coordinator. Clinicians can request additional assessments to be completed by the participants independently before home visits if participants have own device to do so. Participants can also complete additional assessments during their home visits using tablets provided by the research team, by asking the participants to log into the Cambian Navigator.

6.8 Outcome Measures

The following measures are mandatory assessments for participation in both Stage 1 (pre-intervention outcome group) and Stage 2 (for both control and intervention groups).

6.8.1 Primary outcome measures

The primary outcomes of our study are the QOL of patients and family caregivers. Patient QOL will be measured using the McGill Quality of Life Questionnaire-Expanded (MQOL-E) Total (summary) score, which is adapted from MQOL-Revised[64, 65]. MQOL-E has 22 items covering physical, psychological, existential, social, feeling like a burden, surroundings, cognition, financial and quality of health care domains. Family caregiver QOL will be measured using the Quality of Life in Life Threatening Illness-Family Carer Version 2 (QOLLTI-Fv2) Total (summary) score [70]. QOLLTI-Fv2 has 17 items that represent 7 domains of relevance to the QOL of family caregivers, including their environment, the state of the patient, their own state, outlook, relationships with others, quality of care, and financial worries.

6.8.2 Secondary outcome measures

Secondary outcomes include patients' and family caregivers' satisfaction with care. These will be measured using the Canadian Health Care Evaluation Project Questionnaire (CANHELP)[74] Lite version[66] (patients' and family caregivers' health care experience). The patient version has 20 items and covers the following domains: Relationship with Doctors, Illness Management, Communication, Decision-Making, Your Well-being, and Overall Satisfaction. The family caregiver version has 21 items representing the following domains: Relationship with Doctors, Characteristics of Doctors and Nurses, Illness Management, Communication and Decision-Making, Your Involvement, and Overall Satisfaction. Three items on relationships with home health professionals adapted from the questions on relationships with doctors are added to both the CANHELP-LITE assessment for patients and for family caregivers to be used in this study.

The domains measured by the MQOL-E and QOLLTI-Fv2 subscales (listed above) are also secondary outcomes. In addition, these questionnaires have added items for direct rating of the quality of their days (good, average, bad) and changes since the last questionnaire completion (6 items for MQOL-E and 11 for QOLLTI-Fv2) to allow direct evaluation of change over time and to further validate these measures.

6.8.3 Other measures

VR-12 (based on a nearly identical to the SF-12) is a widely-used generic PROM which will be included for the measurement of general physical and mental health status and for cost-effectiveness analysis[67].

We will also collect patient demographic information including: gender, age, education, employment status, occupation, family income, ethnic background, Canadian-born (or not), religion, diagnoses and date of diagnosis. Family caregivers will be asked to provide their own demographic information including: gender, age, education, employment status, occupation, family income, ethnic background, Canadian-born (or not), religion, living arrangements, relationship to patient.

Please see Appendix 6 for information about reliability and validity of outcome measures, and Appendix 18 for revised QOL questionnaires and outcome measures used in the study.

6.9 Analytical Plan

6.9.1 Analysis of clinician focus groups and interviews and patient/family caregiver interviews

All focus group data and interview data will be recorded digitally, transcribed verbatim, and analyzed on an ongoing basis using interpretive description.[75] To ensure trustworthiness and rigor, we will employ iterative cycles of engagement with users (credibility), generate an audit trail (confirmability), and detailed

reporting of the context (transferability)).[75, 76] The transcripts from the first focus group will be read and re-read by 3 researchers to generate an initial codebook (shaped to answer the research objectives) and to encourage interrater reliability. The codebook will be refined in an iterative fashion throughout the analysis. Codes will be categorized and, with evolving analysis, themed. In addition, data about QPSS usage will be monitored and tabulated and used to inform the analysis for qualitative data, with the goal to better understand usages of different aspects of the QPSS for care planning and shared decision making. We will store and code all qualitative data into NVivo.

6.9.2 Analysis of outcomes data

6.9.2.1 Sample size justification

For purposes of estimation, if we assume the effect of routine use of the QPSS intervention is equivalent to changing an average day into a good day for patients (based on the MQOL)[77]. Our power analysis has shown that sample sizes of 432 and 158 would be needed to detect small to moderate effect sizes. We propose a larger sample of 540 (180 for each group) for both patients and family caregivers to account for attrition and a small to moderate effect. Accordingly, each group (pre-intervention, control, intervention) will consist of a total of 180 patients and 180 family caregivers.

Quantitative data will be analyzed using descriptive statistics to describe the pre-intervention comparator sample for patients and family caregivers. Comparative analyses will be completed as part of stage 2 of the project. Following standard descriptive statistical analyses, the effect of the intervention will be assessed based on an intention to treat analysis, as recommended by CONSORT.[78, 79]] All tests will be two-tailed with an alpha level of 0.05. The analysis will focus on the comparison of trajectories of the outcome measures across the randomized treatment and usual care groups stratified by home care office. The area under the curve (AUC) will be calculated for each trajectory (up to six times of administration per participant) to create a summary score. This robust approach is recommended when the goal is to arrive at an overall summary measure over a period of time and when unsystematic fluctuations between measurement points are anticipated (which are not readily captured by fitting a model-based trajectory).[80, 81] Analysis of covariance will be used as the primary method of analysis to compare AUC scores of both outcomes (QOL and health care experience) across the intervention and control groups while controlling for within- and between-group differences (clustered by home care office). A limitation of this approach is that the shapes of the trajectories are not taken into account. We will therefore supplement the primary analysis using (a) graphical displays to visualize the trajectories and (b) individual growth modeling to ascertain whether characteristics of the shapes of the trajectories (slope and curvatures) are different for the three groups.[82-84] Differential item functioning analyses will be conducted on the outcome measures to examine potential gender bias in measurement.[85, 86] Additionally, biological sex will be included as a moderator in the hypothesis testing. Descriptive statistics will be used to describe the use of interventions by clinicians to address identified areas of patient and family caregiver needs.

6.9.3 Analysis of administrative data in cost-effectiveness evaluation

The primary economic evaluation work will involve the comparison of data from stages 1 and 2; the cost-effectiveness evaluation will focus on Stage 2 of the project, running in parallel with, and drawing data from, other components. The framework for the economic analysis will be cost-consequence analysis, where the costs and outcomes of the alternatives are listed separately in a disaggregated format (e.g., intervention costs, hospital costs, clinical benefits, and QOL). As a complement to the cost-consequence analysis, a cost-utility approach will also be employed, with outcomes expressed as quality-adjusted life years (QALYs). The time horizon will be 12 months (consistent with outcomes), and a broad perspective will be adopted to consider both health sector and participant/family costs.

Using health administrative data for each participant, we will determine: (a) frequency and length of use of the QPSS of the home care team, including the number of home care visits and consultations with interdisciplinary team members; and (b) use of other health services, including emergency and acute care admissions. In addition, for the intervention group, the amount of clinician time to use the QPSS and other implementation costs will be captured by the Cambian Coordinator system). The dollar value of resources will be determined using local Fraser Health accounts data, supplemented using the Canadian Institutes of Health Information patient cost estimator for acute sector admissions (www.cihi.ca).

Bootstrapping will be used for comparison of the mean costs and QALYs between groups, with non-parametric confidence intervals, and to estimate the incremental cost-utility ratio.[87, 88] The bootstrapped cost-effect pairs can graphically be represented on a cost-effectiveness plane,[89, 90] and cost-effectiveness acceptability curves calculated, showing the probability that an intervention is cost-effective at a specific threshold ratio.[91, 92] This information will be used to evaluate relative cost for each of the groups and ascertain whether QPSS implementation results in cost-saving or expense.

A data access request will be submitted to identify the specific data elements that will be used for the study.

6.10 Potential Benefits

Some patient and family caregiver participants in our earlier catalyst projects reported that completing the outcome questionnaires stimulated them to reflect on their various areas of their life and care, or normalized how they were feeling, which they found helpful. They may also benefit from the opportunity to potentially improve services to others in their situation.

Clinician participants may not derive any particular benefit from participation in the study; however, they may appreciate the opportunity to have a voice in the development of protocol for implementation of the tablet system into practice. Clinician participants may also be able to gain empowerment from being involved in the development of integrated care for people with life-limiting illness while simultaneously having the opportunity to develop educative-research skills that can support knowledge translation. The clinicians will also gain knowledge and better understanding of the application of the QPSS through the hands-on practice of using the system.

6.11 Potential Risks

6.11.1 Patients and family caregivers

Potential patient and family caregiver participants will be advised that their participation is strictly voluntary and that their decision to participate or not in the study will not compromise the care they are receiving. They will also be advised that they may refuse to answer any questions or withdraw participation in the project at any time. Care providers will be able to see the information from clients and family caregivers about their experiences with care. The intent is for care providers to use this information to provide better care.

6.11.2 Clinicians

No potential for harm is anticipated for clinician participants. Participation in the project will not affect their employment status in any way and their workplace performance will not be evaluated in any way. Clinicians at participating home care offices who choose not to participate will not be affected in any way. Clinician participants will be reminded their participation is voluntary and that they will be able to withdraw from the project at any time without needing to provide an explanation or reason. Any

managers or colleagues who are members of the research team will not have access to any focus group or interview data that includes identifying information.

We recognize that focus group participants may be in supervisor-subordinate relationships, and that participants may not feel free to share all their perspectives as a result. However, the goal is not so much about all the different perspectives but rather about coming to an agreement on adaptations required and how to implement the QPSS in practice. Prior to data collection, participants are reminded that the goal of the focus group is to discuss ways of using the QPSS as fitting with their practice setting, to arrive at an agreed plan that reflects front-line clinicians' and managers' perspectives on the integration of the QPSS in practice.

6.12 Incidental findings

In the event a patient or family caregiver participant has very strong dissatisfaction of care, or is found to be in poor mental health during a phone interview, the research personnel involved in data collection are trained to respond as follows:

- Be empathetic towards the participant's frustrations.
- Explain that the research personnel is a part of Trinity Western University / University of Alberta and suggest that the individual contact Fraser Health (pcqoffice@fraserhealth.ca) or Alberta Health Services to discuss their concerns, so that the issues could be appropriately conveyed to the healthcare administration.
- Ask the participant how he/she can be supported, and provide contacts to the appropriate non-profit agency for support and direction to the participant.
- Reschedule the phone interview, if the participant wishes to do so.
- Inform the project coordinator of the incidental finding.

6.13 Subject Safety Provisions

If the patients and family caregivers disclose thoughts of harm to oneself or to others during the course of the data collection, the research staff is required by law to report the information to the treating team in order to allow for appropriate treatment and follow-up.

Data collection will be stopped at any time upon request by the participants. The provisions for termination of data collection are specified in all participant consent forms.

6.14 Data Security and Protection of Privacy

6.14.1 Focus group (clinician) and interview (clinician, patient, family caregiver) data

Given the location of the focus groups, there are limits to confidentiality regarding clinician participant attendance. By the nature of focus groups, researchers will be aware of the identities of participants in the clinician focus groups. This information will be kept confidential and will not be accessible to anyone outside of the research team. Participants within each focus group will be aware of each other's' identities and of the data provided. At the start of the focus group, we ask that all participants refrain from using names or identifying details of their patients and to be considerate of the privacy of the other participants in the focus group. We further ask that participants do not share what was discussed at the focus group, content and names, with those outside the group. Identities of the participants will be kept confidential and will not be accessible to anyone outside of the research team.

The focus groups (clinicians) and interviews (clinicians, patients, family caregivers) will be digitally audio-recorded and the audio files subsequently transferred from the recording device to a password protected and encrypted computer. The digital recording will then be submitted by the Principal Investigator (PI) or

the research staff to a transcription service (https://www.pointswesttranscription.com/) via a secure link provided by the service. The focus group and interview data will be anonymized during transcription. Transcriptions will be returned via a secure password-protected link. Please see Appendix 21 confirming the confidentiality agreement with the transcription service. This is the same service that has been used for previous Fraser Health research projects. Identifiable information of participants will only be accessible to the PI, Co-Investigators (CIs), research staff and transcriptionist(s). All staff will be made aware of their responsibilities concerning privacy and confidentiality by the PI and CIs.

6.14.2 Patient and family caregiver QPSS data

Patients and family caregivers will be assigned a unique study number upon receipt of their signed consent form. The unique study number is for research purposes and participants do not need to know the number to participate in the study.

As is described in detail in the privacy impact assessment documentation, the Cambian Navigator web application enables patients to complete the questionnaires as per the study protocols. The patient is prompted to share the completed questionnaires with the requesting party (clinicians and/or researchers) and explicit consent is always obtained before the data can be shared. This ensures that consent is always obtained, and only the right information is shared with the various parties.

The web application implements technical, physical, and administrative safeguards to protect against reasonably foreseeable risks to the security, confidentiality, and integrity of personal health information. The system complies with the requirements contained in parts 164.308 (Administrative Safeguards), 164.310 (Physical Safeguards), 164.312 (Technical Safeguards), and 164.316 (Policies and Procedures) of the HIPAA Security Rule. In addition, the data is hosted in Canada and all security services (e.g., intrusion detection) are also performed in Canada.

6.14.3 Data Access

The web application provides role-based access to the system. Accounts can be accessed via computer or tablet.

a) Patients and family caregivers: Each participating patient has an account on the Cambian Navigator system where they can enter, review, and share information with their care providers.
b) Clinicians: Each participating clinician has an account on the Cambian Coordinator system where they can review and analyze information that has been shared with them by patients and family caregivers.
c) Researchers: Each participating researcher has an account on the Cambian Coordinator system where they can review and analyze information that has been shared with them by patients and caregivers.
d) Fraser Health Program Managers: Each participating Fraser Health Program Manager has an account on the Cambian Coordinator system where they can review and analyze information that has been shared with them by patients.
e) Cambian Operations: Support staff at Cambian have administrative access to Cambian Navigator and Cambian Coordinator systems where they can manage operations for these online applications including all typical processes involved in the development and operations of web-based applications.

A Security Threat Risk Assessment has been submitted to assess the architecture of the system and approval has been granted.

6.14.4 Storage of paper and audio data

All original paper-and audio-based data will be stored in locked filing cabinets in locked offices and on TWU-owned / U of A-owned password protected and encrypted computers. Data that includes any personally identifiable information will not be shared among team members using email or other insecure data transfer methods. The study code key that links the ID to the identity of each study participant in the outcomes evaluation will be saved separately on a secure server at TWU for the duration of the project. The PI is responsible for the deletion of the study code key document from TWU server at the conclusion of the study. All directly identifying participants' information will be shredded and electronic files will be deleted when the study is completed. Study data will be retained indefinitely after completion of the study. The principal investigator will ultimately be responsible for all data monitoring, analysis, and disposal.

6.15 Ethics Approval

Ethics approval will be sought from the Fraser Health Research Ethics Board, the Trinity Western University, University of Victoria, Simon Fraser University, University of British Columbia, University of Alberta and Alberta Health Services Research Ethics Board. Once approval is obtained by the local university and health authority, the study will be conducted in accordance with the respective Research Ethics Boards' policy.

7 PLANS FOR PUBLICATION AND CONFERENCE PRESENTATIONS

Study findings will be presented at universities and health authority seminars, regional and international conferences, education forums and professional rounds. Manuscripts will be submitted to journals identified to be of high relevance to the research topic.

8 TIMELINE

Enrolment of participating home care offices began in February 2016, starting at 2 Fraser Health home care offices: Tri-cities and New Westminster. Two additional Fraser Health home care offices- Burnaby and Gateway- were enrolled in July/August 2016; another 2 offices - White Rock and South Delta- were subsequently enrolled in December 2016. Enrolment of Alberta Health Services Edmonton Zone Home Living was negotiated before the end of 2016. The current amendment of the study protocol is for all Fraser Health home care offices and Alberta Health Services Edmonton Zone enrolled in this study. Data collection for the focus groups and baseline data for outcomes evaluation will be carried out over the course of 12 months. Interviews with patients and family caregivers will be conducted during months 7 to 12 of Stage 1 and over 1 year in Stage 2 at each home care office. Feedback from clinicians about their experience of using the QPSS with patients and family caregivers will be provided during the focus groups and interviews in Stage 1 transition phase and Stage 2 of the study.

9 REFERENCES

1. World Health Association. *WHO Definition of Palliative Care.* 2012 June 6, 2012]; Available from: http://whqlibdoc.who.int/hist/official_records/2e.pdf.
2. Cohen, S.R. and B.M. Mount, Pain with life-threatening illness: Its perception and control are inextricably linked with quality of life. Pain Research & Management, 2000. **5**(4): p. 271-275.
3. National Institute for Clinical Excellence, Guidance on cancer services: Improving supportive and pallaitive care for adults with cancer: The manual. 2004, NICE: London.
4. Cohen, S.R. and A. Leis, What determines the quality of life of terminally ill cancer patients from their own perspective? Journal of Palliative Care, 2002. **18**(1): p. 48-58.
5. Grande, G. and G. Ewing, Death at home unlikely if informal carers prefer otherwise: Implications for policy. Palliative Medicine, 2008. **22**(8): p. 971-2.
6. McCorkle, R., et al., The effects of home nursing care for patients during terminal illness on the bereaved's psychological distress. Nursing Research, 1998. **47**(1): p. 2-10.
7. Luker, K.A., et al., The importance of 'knowing the patient': Community nurses' constructions of quality in providing palliative care. Journal of Advanced Nursing, 2000. **31**(4): p. 775-82.
8. Gomes, B. and I.J. Higginson, Factors influencing death at home in terminally ill patients with cancer: Systematic review. British Medical Journal, 2006. **332**(7540): p. 515-21.
9. Stajduhar, K.I. and B. Davies, Variations in and factors influencing family members' decisions for palliative home care. Palliative Medicine, 2005. **19**(1): p. 21-32.
10. Aoun, S.M., et al., *Caregiving for the terminally ill: At what cost?* Palliative Medicine, 2005. **19**(7): p. 551-5.
11. Schulz, R. and S.R. Beach, *Caregiving as a risk factor for mortality: The Caregiver Health Effects Study.* Journal of the American Medical Association, 1999. **282**(23): p. 2215-9.
12. Funk, L., et al., Part 2: Home-based family caregiving at the end of life: a comprehensive review of published qualitative research (1998-2008). Palliative medicine, 2010. **24**(6): p. 594-607.
13. Donaldson, M.S., Taking PROs and patient-centered care seriously: Incremental and disruptive ideas for incorporating PROs in oncology practice. Quality of Life Research, 2008. **17**(10): p. 1323-30.
14. Greenhalgh, J., The applications of PROs in clinical practice: What are they, do they work, and why? Quality of Life Research, 2009. **18**(1): p. 115-23.
15. Lohr, K.N. and B.J. Zebrack, *Using patient-reported outcomes in clinical practice: Challenges and opportunities.* Quality of Life Research, 2009. **18**(1): p. 99-107.
16. Marshall, S., K. Haywood, and R. Fitzpatrick, *Impact of patient-reported outcome measures on routine practice: A structured review.* Journal of Evaluation in Clinical Practice, 2006. **12**(5): p. 559-68.
17. Osoba, D., *Translating the science of patient-reported outcomes assessment into clinical practice.* Journal of the National Cancer Institute. Monographs, 2007(37): p. 5-11.
18. Rose, M. and A. Bezjak, Logistics of collecting patient-reported outcomes (PROs) in clinical practice: An overview and practical examples. Quality of Life Research, 2009. **18**(1): p. 125-36.
19. Valderas, J.M., et al., The impact of measuring patient-reported outcomes in clinical practice: A systematic review of the literature. Quality of Life Research, 2008. **17**(2): p. 179-93.
20. Velikova, G., et al., Measuring quality of life in routine oncology practice improves communication and patient well-being: A randomized controlled trial. Journal of Clinical Oncology, 2004. **22**(4): p. 714-24.
21. Cooper, K., *Getting the measure of the patient experience.* Nursing Times, 2013. **109**(23): p. 12-4.
22. Jenkinson, C., A. Coulter, and S. Bruster, The Picker Patient Experience Questionnaire: development and validation using data from in-patient surveys in five countries. International Journal for Quality in Health Care, 2002. **14**(5): p. 353-8.
23. Jenkinson, C., et al., Patients' experiences and satisfaction with health care: Results of a questionnaire study of specific aspects of care. Quality & Safety in Health Care, 2002. **11**(4): p. 335-9.
24. Aktas, A., et al., Connected Health: Cancer Symptom and Quality-of-Life Assessment Using a Tablet Computer: A Pilot Study. Am J Hosp Palliat Care, 2013.

25. Schwartz, C.E., et al., Evaluation of the Missoula-VITAS Quality of Life Index--revised: Research tool or clinical tool? Journal of Palliative Medicine, 2005. **8**(1): p. 121-35.
26. Taenzer, P.A., et al., *Computerized quality-of-life screening in an oncology clinic.* Cancer Practice, 1997. **5**(3): p. 168-75.
27. Santana, M.J. and D.H. Feeny, Using the health utilities index in routine clinical care: Process, feasibility, and acceptability: A randomized controlled trial. Patient, 2009. **2**(3): p. 159-67.
28. Hilarius, D.L., et al., Use of health-related quality-of-life assessments in daily clinical oncology nursing practice: A community hospital-based intervention study. Cancer, 2008. **113**(3): p. 628-37.
29. Greenhalgh, J. and K. Meadows, The effectiveness of the use of patient-based measures of health in routine practice in improving the process and outcomes of patient care: A literature review. Journal of Evaluation in Clinical Practice, 1999. **5**(4): p. 401-16.
30. Dunckley, M., et al., *A research study to identify facilitators and barriers to outcome measure implementation.* International Journal of Palliative Nursing, 2005. **11**(5): p. 218-25.
31. Eischens, M.J., B.A. Elliott, and T.E. Elliott, *Two hospice quality of life surveys: A comparison.* The American Journal of Hospice & Palliative Care, 1998. **15**(3): p. 143-8.
32. Hill, N., Use of quality-of-life scores in care planning in a hospice setting: A comparative study. International Journal of Palliative Nursing, 2002. **8**(11): p. 540-7.
33. Pratheepawanit, N., M.S. Salek, and I.G. Finlay, The applicability of quality-of-life assessment in palliative care: Comparing two quality-of-life measures. Palliative Medicine, 1999. **13**(4): p. 325-34.
34. Mullen, K.H., D.L. Berry, and B.K. Zierler, Computerized symptom and quality-of-life assessment for patients with cancer part II: Acceptability and usability. Oncology Nursing Forum, 2004. **31**(5): p. E84-896.
35. Suh, S.Y., et al., Longitudinal patient-reported performance status assessment in the cancer clinic is feasible and prognostic. Journal of Oncology Practice, 2011. **7**(6): p. 374-81.
36. Black, N., Patient reported outcome measures could help transform healthcare. BMJ, 2013. **346**: p. f167.
37. Devlin, N.J. and J. Appleby, Getting the most out of proms. Putting health outcomes at the heart of NHS decision-making. 2010, The King's Fund.
38. Feeny, D., Health-related quality-of-life data should be regarded as a vital sign. J Clin Epidemiol, 2013. **66**(7): p. 706-9.
39. Harding, R. and I. Higginson, *Working with ambivalence: informal caregivers of patients at the end of life.* Support Care Cancer, 2001. **9**(8): p. 642-5.
40. Abernethy, A.P., et al., Feasibility and acceptability to patients of a longitudinal system for evaluating cancer-related symptoms and quality of life: Pilot study of an e/tablet data-collection system in academic oncology. Journal of Pain & Symptom Management, 2009. **37**(6): p. 1027-1038.
41. Abernethy, A.P., et al., Improving health care efficiency and quality using tablet personal computers to collect research-quality, patient-reported data. Health Services Research, 2008. **43**(6): p. 1975-1991.
42. Basch, E. and A.P. Abernethy, *Supporting clinical practice decisions with real-time patient-reported outcomes.* Journal of Clinical Oncology, 2011. **29**(8): p. 954-6.
43. Berry, D.L., et al., Enhancing patient-provider communication with the electronic self-report assessment for cancer: A randomized trial. Journal of Clinical Oncology, 2011. **29**(8): p. 1029-1035.
44. Berry, D.L., et al., Computerized aymptom and quality-of-life assessment for patients with cancer part I: Development and pilot testing. Oncology Nursing Forum, 2004. **31**(5): p. E75-895.
45. Rogausch, A., et al., Feasibility and acceptance of electronic quality of life assessment in general practice: An implementation study. Health & Quality of Life Outcomes, 2009. **7**: p. 1-11.
46. Taenzer, P., et al., Impact of computerized quality of life screening on physician behaviour and patient satisfaction in lung cancer outpatients. Psycho-Oncology, 2000. **9**(3): p. 203-213.
47. Dupont, A., et al., *Use of tablet personal computers for sensitive patient-reported information.* The Journal of Supportive Oncology, 2009. **7**(3): p. 91-7.

48. Bates, D.W., et al., Ten commandments for effective clinical decision support: Making the practice of evidence-based medicine a reality. Journal of the American Medical Association, 2003. **10**(6): p. 523-30.
49. Detmar, S.B., et al., Health-related quality-of-life assessments and patient-physician communication: A randomized controlled trial. Journal of the American Medical Association, 2002. **288**(23): p. 3027-34.
50. Hughes, R., et al., *Professionals' views and experiences of using outcome measures in palliative care.* International Journal of Palliative Nursing, 2003. **9**(6): p. 234-8.
51. Antunes, B., R. Harding, and I.J. Higginson, Implementing patient-reported outcome measures in palliative care clinical practice: A systematic review of facilitators and barriers. Palliat Med, 2014. **28**(2): p. 158-75.
52. Bruera, E., et al., The Edmonton Symptom Assessment System (ESAS): A simple method for the assessment of palliative care patients. Journal of Palliative Care, 1991. **7**(2): p. 6-9.
53. Watanabe, S., et al., *The Edmonton symptom assessment system--what do patients think?* Support Care Cancer, 2009. **17**(6): p. 675-83.
54. Watanabe, S.M., et al., A multicenter study comparing two numerical versions of the Edmonton Symptom Assessment System in palliative care patients. Journal of Pain and Symptom Management, 2011. **41**(2): p. 456-68.
55. Barnett, J. and A. Syme, Adopting and introducing new technology to improve patient care: a wedding of clinicians and informatics specialists. Stud Health Technol Inform, 2009. **143**: p. 343-7.
56. Etkind, S.N., et al., Capture, Transfer, and Feedback of Patient-Centered Outcomes Data in Palliative Care Populations: Does It Make a Difference? A Systematic Review. Journal of Pain and symptom Management, First published online: 2014).
57. Catania, G., et al., Effectiveness of complex interventions focused on quality-of-life assessment to improve palliative care patients' outcomes: A systematic review. Palliative Medicine, First published online: 2014.
58. Campbell, N.C., et al., *Designing and evaluating complex interventions to improve health care.* British Medical Journal, 2007. **334**(7591): p. 455-9.
59. Craig, P., et al., Developing and evaluating complex interventions: the new Medical Research Council guidance. British Medical Journal, 2008. **337**: p. a1655.
60. Catania, G., et al., Does quality of life assessment in palliative care look like a complex screening program? Health and Quality of Life Outcomes, 2013. **11**.
61. Graham, I. and J. Tetroe, *The knowledge to action framework*, in *Models and frameworks for implementing evidence-based practice: Linking evidence to action.*, J. Rycroft-Malone and T. Bucknall, Editors. 2010, Wiley-Blackwell: Oxford, UK. p. 207-221.
62. Straus, S.E., J. Tetroe, and I.D. Graham, *Knowledge translation in health care: Moving from evidence to practice*. 2009, Chichester, West Sussex, UK: Wiley-Blackwell. xvii, 318 p.
63. Sawatzky, R., et al. *Patient- and Family-Reported Experience and Outcome Measures for Use in Acute Care: A Knowledge Synthesis*. 2015 2015/05/01]; Available from: http://figshare.com/articles/PATIENT_AND_FAMILY_REPORTED/1412721.
64. Cohen, S.R., et al., Validity of the McGill Quality of Life Questionnaire in the palliative care setting: A multi-centre Canadian study demonstrating the importance of the existential domain. Palliative Medicine, 1997. **11**(1): p. 3-20.
65. Cohen, S., et al. Two new versions of the McGill Quality of Life Questionnaire (MQOL). in Oral presentation at the 14th World Congress of the European Association of Palliative Care. May 8-10th, 2015. . 2015. Copenhagen, Denmark. : European Journal of Palliative Care Suppl. .
66. Heyland, D.K., et al., The development and validation of a novel questionnaire to measure patient and family satisfaction with end-of-life care: The Canadian Health Care Evaluation Project (CANHELP) Questionnaire. Palliative Medicine, 2010. **24**(7): p. 682-95.

67. Iqbal, S.U., et al., *The Veterans RAND 12 Item Health Survey (VR-12): what it is and how it is used.* Washington, DC: Veterans Health Administration, 2009.
68. Davison, S.N., G.S. Jhangri, and J.A. Johnson, *Longitudinal validation of a modified Edmonton symptom assessment system (ESAS) in haemodialysis patients.* Nephrology, Dialysis, Transplantation, 2006. **21**(11): p. 3189-95.
69. Davison, S.N., G.S. Jhangri, and J.A. Johnson, Cross-sectional validity of a modified Edmonton symptom assessment system in dialysis patients: A simple assessment of symptom burden. Kidney International, 2006. **69**(9): p. 1621-5.
70. Cohen, R., et al., *QOLLTI-F: Measuring family carer quality of life.* Palliative Medicine, 2006. **20**(8): p. 755-67.
71. Ewing, G., et al., The Carer Support Needs Assessment Tool (CSNAT) for Use in Palliative and End-of-life Care at Home: A Validation Study. J Pain Symptom Manage, 2012.
72. Ewing, G. and G. Grande, Development of a Carer Support Needs Assessment Tool (CSNAT) for end-of-life care practice at home: A qualitative study. Palliative Medicine, 2013. **27**(3): p. 244-56.
73. Dillman, D.A., J.D. Smyth, and L.M. Christian, *Internet, phone, mail, and mixed-mode surveys : the tailored design method*. 4th edition. ed. 2014, Hoboken: Wiley. xvii, 509 pages.
74. Heyland, D.K., et al., The development and validation of a shorter version of the Canadian Health Care Evaluation Project Questionnaire (CANHELP Lite): A novel tool to measure of Patient and Family Satisfaction with End of Life Care. Journal of Pain and Symptom Management, in press.
75. Thorne, S., *Interpretative description*. 2008, Walnut Creek, CA: Left Coast Press.
76. Lincoln, Y.S. and E.G. Guba, *Naturalistic Inquiry*. 1985, Beverly Hills, CA: Sage Publications.
77. Cohen, S.R. and B.M. Mount, Living with cancer: "good" days and "bad" days--what produces them? Can the McGill quality of life questionnaire distinguish between them? Cancer, 2000. **89**(8): p. 1854-65.
78. Polit, D.F. and B.M. Gillespie, Intention-to-treat in randomized controlled trials: recommendations for a total trial strategy. Res Nurs Health, 2010. **33**(4): p. 355-68.
79. Schulz, K.F., D.G. Altman, and D. Moher, CONSORT 2010 Statement: updated guidelines for reporting parallel group randomised trials. BMC Med, 2010. **8**: p. 18.
80. Fayers, P., Applying item response theory and computer adaptive testing: the challenges for health outcomes assessment. Quality of Life Research, 2007. **16**(Suppl 1): p. 187-94.
81. Walters, S.J., Quality of life outcomes in clinical trials and health-care evaluation : a practical guide to analysis and interpretation. Statistics in practice. 2009, Chichester, West Sussex, U.K.: Wiley. xii, 365 p.
82. Heck, R.H., S. Thomas, and L.N. Tabata, *Multilevel modeling of categorical outcomes using IBM SPSS*. Quantitative methodology series. 2012, New York ; London: Routledge. xvi, 439 p.
83. Shek, D.T. and C.M. Ma, Longitudinal data analyses using linear mixed models in SPSS: Concepts, procedures and illustrations. ScientificWorldJournal, 2011. **11**: p. 42-76.
84. Chen, H. and P. Cohen, Using individual growth model to analyze the change in quality of life from adolescence to adulthood. Health Qual Life Outcomes, 2006. **4**: p. 10.
85. Scott, N.W., et al., Differential item functioning (DIF) analyses of health-related quality of life instruments using logistic regression. Health and Quality of Life Outcomes, 2010. **8**: p. 81.
86. Teresi, J.A., Different approaches to differential item functioning in health applications. Advantages, disadvantages and some neglected topics. Med Care, 2006. **44**(11 Suppl 3): p. S152-70.
87. Barber, J.A. and S.G. Thompson, Analysis of cost data in randomized trials: an application of the non-parametric bootstrap. Stat Med, 2000. **19**(23): p. 3219-36.
88. Briggs, A.H. and A.M. Gray, Handling uncertainty when performing economic evaluation of healthcare interventions. Health Technol Assess, 1999. **3**(2): p. 1-134.
89. Korthals-de Bos, I., et al., Economic evaluations and randomized trials in spinal disorders: principles and methods. Spine (Phila Pa 1976), 2004. **29**(4): p. 442-8.
90. Black, W.C., The CE plane: a graphic representation of cost-effectiveness. Med Decis Making, 1990. **10**(3): p. 212-4.

91. Fenwick, E., K. Claxton, and M. Sculpher, *Representing uncertainty: the role of cost-effectiveness acceptability curves.* Health Econ, 2001. **10**(8): p. 779-87.
92. Fenwick, E., B.J. O'Brien, and A. Briggs, Cost-effectiveness acceptability curves--facts, fallacies and frequently asked questions. Health Econ, 2004. **13**(5): p. 405-15.
93. Chang, V.T., S.S. Hwang, and M. Feuerman, *Validation of the Edmonton Symptom Assessment Scale.* Cancer, 2000. **88**(9): p. 2164-71.
94. Cohen, S.R., et al., Quality of life in HIV disease as measured by the McGill Quality of Life Questionnaire. AIDS, 1996. **10**(12): p. 1421-1427.
95. Cohen, S.R., et al., The McGill Quality of Life Questionnaire: A measure of quality of life appropriate for people with advanced disease. A preliminary study of validity and acceptability. Palliative Medicine, 1995. **9**(3): p. 207-19.
96. Gandek, B., et al., Cross-validation of item selection and scoring for the SF-12 Health Survey in nine countries: results from the IQOLA Project. International Quality of Life Assessment. J Clin Epidemiol, 1998. **51**(11): p. 1171-8.
97. Ware, J., Jr., M. Kosinski, and S.D. Keller, A 12-Item Short-Form Health Survey: construction of scales and preliminary tests of reliability and validity. Med Care, 1996. **34**(3): p. 220-33.
