# Supplementary material for: Did a digital quality of life (QOL) assessment and practice support system in home health care improve the QOL of older adults living with life-limiting conditions and of their family caregivers? A mixed-methods pragmatic randomized controlled trial
Source: PLoS One. 2025 May 6;20(5):e0320306. doi: 10.1371/journal.pone.0320306 (PMC12054893; doi:10.1371/journal.pone.0320306)
Supplement: Attachment — Submitted filename: Response to Reviewers PONE-D-24-23372.docx [file pone.0320306.s008.docx]

**PLOS ONE Manuscript** **PONE-D-24-23372:**

Did a digital quality of life (QOL) assessment and practice support system in home health care improve the QOL of older adults living with life-limiting conditions and of their family caregivers?

A mixed-methods pragmatic randomized controlled trial.

**Response to Reviewers’ Comments**

The comments from the three reviewers were very helpful – thank you.

As requested, this is our “rebuttal letter that responds to each point raised by the academic editor and reviewers.”

We have addressed each comment in the table below. Please note that the line numbers correspond to the version of the manuscript without tracked changes.

| **Academic Editor** | **Response** | **Line** |
| --- | --- | --- |
| 1. Please ensure that your manuscript meets PLOS ONE's style requirements, including those for file naming. The PLOS ONE style templates can be found at  <https://journals.plos.org/plosone/s/file?id=wjVg/PLOSOne_formatting_sample_main_body.pdf> and  <https://journals.plos.org/plosone/s/file?id=ba62/PLOSOne_formatting_sample_title_authors_affiliations.pdf> | We have checked and revised throughout. |  |
| **Reviewer #1** | **Response** | **Line** |
| 1. What are the benefits of using SEM over linear mixed models (LMM) or Generalized Estimating Equations (GEE), which are generally easier to understand and interpret? LMM directly estimates group means and slopes and GEE provides flexibility in handling violations of normality. | We acknowledge that both LMM and GEE are relatively simple yet robust approaches for estimating population averaged effects. However, having observed substantial heterogeneity, we were explicitly interested in modeling individual-level variation. We also observed that the individual trajectories were not linear, nor did we have a hypothesis of any particular parametric shape. Further, given the observed missing data patterns, we elected to employ models that would allow for comparisons of different missing data assumptions. Based on these premises, we decided to use the level and shape specification for a longitudinal structural equation model. We added several sentences to explain our choice of utilizing this model. | 347-354 |
| 2. Add more demographic /baseline information, e.g. Age, and chronic conditions. | This information is included in the results and in Fig 5, where we provide information about age, gender, marital status, family income, highest level of education, family income, living arrangement, country of birth, relationship between patient and family caregiver, ethnic background, self-reported diagnoses for patients. The supplementary materials provide additional details about the demographic and baseline information for patients (S1 Table), family caregivers (S2 Table), and clinician participants (S3 Table). No changes have been made in response to this comment. | 411-426  Fig 5  S1 Table  S2 Table  S3 Table |
| 3. Figure 4: Allocation (n=???) | Thank you. For some reason the n was hidden in the figure. This has now been corrected (n = 373). | Fig 4 |
| **Reviewer #2** | **Response** | **Line** |
| 1. This is a very important paper about use of PROMs by home care teams. This is such a through report on methods and processes. Typically the reporting of methods such that interventions cannot be replicated based on report is the primary critique in reviews. Thank you for the superior description of the intervention. … Findings again are well reported. | Thank you. This was part of our motivation for submitting to this journal. |  |
| On page 21, you end with "Negative case analysis revealed that there were alternative perspectives to each of these explanations." You really leave your reader hanging. Can you provide some continuity? | Thank you for pointing this out. This information was embedded in the supplementary Table 4. We have now expanded the corresponding paragraph in the text to explicitly identify the negative case analysis results with example quotes for action phase E. We also added a sentence to the previous section on action phases B-D. | 468-469  492-504 |
| **Discussion** | | |
| 2. I would like to see more discussion about the use of the Carer Support Needs Assessment Tool Intervention. It is NOT meant to be used as a scale or checklist but as a conversation starter. The mean short scan of 24 seconds is disappointing. It was used in a study of changing needs over time in the transplant literature and certainly did show how needs changed over time. I suspect there may be a worthwhile paper on that from this research. | We used the CSNAT that was available at the time this study was planned with the intention of informing the clinicians about the clients’ needs. We did not use the CSNAT as a scale or a checklist and intentionally did not include the CSNAT responses in the modeling. We offered these data to the clinicians with the belief that they would undertake a process of assessment and intervention in a manner comparable to what later became known as the CSNAT intervention. We cannot account for the minimal time spent by the clinicians and agree that this is worthy of future research. Unfortunately, we do not have the data to further explore this. We have added two columns to Table 1 about the original purpose of the assessment tools and how they were used in our study. In the discussion, we now refer to the OSCARSS trial of the CSNAT as an example of similar challenges. | Table 1  581-588 |
| 3. The other piece that I think where more discussion would be useful is on diversity -- caregivers and clients (spagetti plots). Discussion might be fruitful on the lack of person-centered care because with such diversity, in my view care needs to be person-centered in order to have any impact on outcomes such as quality of life or anxiety/ burden. | We recognize there are many possible explanations for the heterogeneity in QOL trajectories. However, we do not have evidence to make a claim about person-centredness, given that we did not explicitly measure this or ask about this in the interviews. Instead, we recommend that future studies should seek to explain heterogeneity in QOL trajectories. We added this recommendation to the discussion. (We are undertaking a related study to further investigate heterogeneity QOL trajectories.) | 635-639 |
| 4. Another area that would be useful to understand is why the cursory use of the added information. Were they able to tailor services based on the information in the assessments? What incentives did they have to use the new information? | We agree. It was rather unexpected that so little time was spent with the QOL assessment information, given the enthusiasm of healthcare providers during the initial stages of the study and the support provided by the healthcare administrators. Unfortunately, we did not collect data to further investigate this. We added text to the discussion about several possible explanations. | 586-595  602-605 |
| 5. All in all this is a paper that needs to be published. I would just like as fulsome a discussion as you had with methods. To me, there was not enough of a so-what to improve home care in the discussion.  This is excellent research and an excellent report. | We agree there is great need for further research to better understand how to improve homecare and the factors that influence implementation of digital QOL assessment tools. Although we did not have the data to further explore this, we have now added text to the discussion and a reference to a recent systematic review on facilitators and barriers of implementation to draw attention to this. | 579-588  594-597 |
| **Reviewer #3** | **Response** | **Page** |
| 1. Discuss strategies that could be employed to minimize loss to follow-up. What proactive measures were taken to maintain participant engagement throughout the study? | We have elaborated that the QOL assessment and data collection procedures involved an invitation for an in-person initial home visit by research staff followed by an extensive schedule of regular reminders and personalized modes of completion of the assessments online, on paper, by phone, or in person. Despite this extensive engagement, and participants recognizing the importance of this study, this is a difficult population to retain due to the life-limiting and progressive nature of the health conditions. | 182-191 |
| 2. Can the authors clarify their choice of statistical methods for analyzing QOL data? Were any alternative methods considered that could account for heterogeneity in QOL trajectories? | We have elaborated on this. Please see our response to reviewer #1. | 347-354 |
| 3. Consider providing a more explicit summary of the key findings at the beginning of the discussion. This would help orient readers before delving into interpretations and implications. | We made a modification to the introductory paragraph of the discussion to make this more explicit. | 570-574 |
| 4. You mention clinician engagement with the QPSS was limited. What strategies do you propose to enhance this engagement in future studies? | Given that we followed the knowledge-to-action cycle to implement established guidelines and recommendations for integrating QOL information into daily practice, and considering the enthusiasm of the clinicians, we were indeed surprised that the clinicians were not more engaged with the QPSS. Our study was not designed to examine this further. To address your comment, we have now added general recommendations from recent literature reviews to the discussion section on facilitators and barriers that may influence the implementation of digital QOL assessments. | 579-588  594-597 |
| 5. Have you considered external factors (e.g., changes in healthcare policies, community resources) that may have influenced the QOL outcomes during the study? How might these be controlled for in future research? | Given that our study was designed as a pragmatic randomized controlled trial, factors influencing the QOL outcomes were controlled for by means of randomization. Nonetheless, it is important to consider that a pragmatic trial involves conducting the research in real-world populations and settings. We have now added a brief clarification about this in parentheses to the first sentence of the design section. Given the pragmatic nature of the trial, it is quite possible that external factors may have influenced the extent to which home healthcare teams used the QPSS. However, other than ongoing restructuring of home care service delivery (as described in the manuscript), we were not aware of any factors of an extraordinary nature while the study was in progress. | 139-142 |
| 6. Consider highlighting specific recommendations for practice based on the findings. | Given the results, we do not have a basis for highlighting practice recommendations. We have therefore instead provided recommendations for future research. Additionally, we have now added further guidance from the literature on facilitators and barriers that may influence the implementation of digital QOL assessments (see comments above). | 631-649 |
