# Supplementary material for: Did a digital quality of life (QOL) assessment and practice support system in home health care improve the QOL of older adults living with life-limiting conditions and of their family caregivers? A mixed-methods pragmatic randomized controlled trial
Source: PLoS One. 2025 May 6;20(5):e0320306. doi: 10.1371/journal.pone.0320306 (PMC12054893; doi:10.1371/journal.pone.0320306)
Supplement: Attachment — Submitted filename: Response_to_Reviewers_PONE-D-24-23372_auresp_2.docx [file pone.0320306.s009.docx]

**PLOS ONE Manuscript** **PONE-D-24-23372:**

Did a digital quality of life (QOL) assessment and practice support system in home health care improve the QOL of older adults living with life-limiting conditions and of their family caregivers?

A mixed-methods pragmatic randomized controlled trial.

**Response to Reviewers’ Comments**

As requested, this is our “rebuttal letter that responds to each point raised by the academic editor and reviewers.”

We have addressed each comment in the table below.

| **Reviewer #1** | **Response** | **Line** |
| --- | --- | --- |
| 1. LMM and GEE are simpler and more flexible approaches for handling repeated measures. You can treat time points as categorical variables, reducing the need to specify a linear or nonlinear parametric pattern. Individual variability can also be explained with variance-covariance matrix setup. Furthermore, mixed models provide unbiased estimations when data are missing at random, and can handle different missing pattern with imputation. | Thank you for your engagement with our work. Indeed, there many modeling approaches that could be used for time series analyses (including multilevel models and generalized estimating equations). While we recognize a desire for ease of interpretation, we believe that our selected method is well-suited to address the objectives of our study. We followed well-established procedures for using longitudinal structural equation models and explained our methodological choices in the statistical analysis second of the manuscript. | No revisions made |
| **Reviewer #2** | **Response** | **Line** |
| 1. I cannot wait to cite this paper. It is excellent. Thank you. In our work and other work with family caregivers, health providers always rate themselves as very good or excellent in their communication and partnership with family caregivers. The conundrum from Miichelle Lobchuk's "In your shoes" communication work that rarely do family caregivers give health providers more than 50% in their first ratings. | Thank you for this encouraging and affirmative comment. | No revisions made |
